# Explainability does not mitigate the negative impact of incorrect AI advice in a personnel selection task

**DOI:** 10.1038/s41598-024-60220-5

**Published:** 2024-04-28

**Authors:** Julia Cecil, Eva Lermer, Matthias F. C. Hudecek, Jan Sauer, Susanne Gaube

**Affiliations:** 1 Department of Psychology, LMU Center for Leadership and People Management, LMU Munich, Munich, Germany; 2 Department of Business Psychology, Technical University of Applied Sciences Augsburg, Augsburg, Germany; 3 Department of Experimental Psychology, University of Regensburg, Regensburg, Germany; 4 Department of Business Administration, University of Applied Sciences Amberg-Weiden, Weiden, Germany; 5 UCL Global Business School for Health, University College London, London, UK

**Keywords:** Artificial intelligence, Personnel selection, Decision-making, Psychology, Human behaviour

## Abstract

Despite the rise of decision support systems enabled by artificial intelligence (AI) in personnel selection, their impact on decision-making processes is largely unknown. Consequently, we conducted five experiments (*N* = 1403 students and Human Resource Management (HRM) employees) investigating how people interact with AI-generated advice in a personnel selection task. In all pre-registered experiments, we presented correct and incorrect advice. In Experiments 1a and 1b, we manipulated the source of the advice (human vs. AI). In Experiments 2a, 2b, and 2c, we further manipulated the type of explainability of AI advice (2a and 2b: heatmaps and 2c: charts). We hypothesized that accurate and explainable advice improves decision-making. The independent variables were regressed on task performance, perceived advice quality and confidence ratings. The results consistently showed that incorrect advice negatively impacted performance, as people failed to dismiss it (i.e., overreliance). Additionally, we found that the effects of source and explainability of advice on the dependent variables were limited. The lack of reduction in participants’ overreliance on inaccurate advice when the systems’ predictions were made more explainable highlights the complexity of human-AI interaction and the need for regulation and quality standards in HRM.

## Introduction

The use of decision support systems enabled by artificial intelligence (AI) has proliferated in various areas, ranging from healthcare to human resource management (HRM^1^). One area in HRM in which AI systems are already used is resume screening in personnel selection^2^, which represents an ideal application of AI due to the sheer amount of available data^3,4^. However, research on the usage of AI systems in HRM is currently limited, and findings have been mixed^5,6^. Given that personnel decisions substantially impact organizational performance^7^ and individuals’ lives, it is crucial to understand how AI-generated advice influences high-stakes decisions and to optimize the interaction between AI systems and their users. This need for a better understanding of the interaction between AI-enabled systems and humans is particularly high when AI algorithms produce advice that humans should consider when making important decisions^8^. Consequently, the willingness to rely on advice plays a pivotal role in integrating AI-generated predictions into human decision-making processes.

It is well-established that people often rely on and are heavily affected by advice^9–11^. When advice is presented, many psychological phenomena come into play: for instance, the anchoring effect^12^, where advice can serve as a starting point (i.e., anchor) from which people make decisions and sometimes fail to adjust sufficiently. Even after an initial decision has been made, advice can lead people to alter their decision toward the given value^8,10^. AI-enabled decision support systems are often used in critical contexts where incorrect adjustments from the advice can have far-reaching consequences, e.g., in medical care or personnel selection. Therefore, it is crucial to understand how different advice characteristics contribute to its adoption and how erroneous reliance can be prevented.

First, the *source of advice* can affect people’s reliance on it. On the one hand, studies across different domains demonstrated a preference for human over AI advice^8,13^, i.e., people reject algorithm advice more often than human advice, even when the human is obviously inferior to the algorithm (*algorithmic aversion*^14^). On the other hand, it has also been shown that individuals are more willing to adhere to algorithmic than human advice (*algorithmic appreciation*^15^). This tendency is particularly evident in tasks with measurable outcomes that require logical problem-solving or in judgments under uncertainty^16^. The underlying mechanisms leading to either algorithmic aversion or appreciation are not yet fully understood, with several advice characteristics proposed as potentially relevant, including the quality of advice. Therefore, we investigated whether we find algorithmic aversion or algorithmic appreciation within a personnel decision-making task.

Second, the *accuracy of advice* also influences the extent to which it is followed. Receiving correct advice leads to higher trust in the advice and improved performance compared to incorrect advice^17–20^. Yet, research has also shown that recipients often blindly follow advice, even when it is incorrect^9,10,17^. Reliance on advice from decision support systems without searching for or ignoring contradictory information is called *automation bias*^21,22^. This *overreliance* on incorrect AI advice by failing to dismiss it may become an increasingly common phenomenon. We, therefore, hypothesized that receiving correct advice (vs. incorrect advice) positively affects decision-making in the personnel selection task.

Research has shown that acceptance and use of AI-enabled systems increase when individuals trust the system’s predictions, which will most likely increase when people regularly interact with these systems and perceive it as useful^23^. One AI-specific characteristic that might impact people’s trust in and reliance on AI advice is its “black-box” nature, which means that the way an algorithm arrives at a result is usually incomprehensible to developers and users^24,25^. Researchers and developers are seeking solutions to overcome this limitation by explaining how and why an algorithm makes a particular prediction. Hence, *explainable AI systems* provide additional information to recipients to make their operations clearer or easier to understand by reducing the model’s complexity or simplifying its outputs^26^. The form in which explanations are presented depends on the algorithm’s task and the user. For instance, for convolutional neural networks (CNNs), commonly used for image classification, explainability is often achieved by highlighting areas on the images that are particularly relevant to the outcome using heatmaps^26^. Visualizations using salient heatmaps are also widely deployed to explain the results of AI algorithms relying on natural language processing (NLP), employed for the analysis of written or spoken language^27–31^. In addition to this comprehensive and rather complex visualization method for explanations, existing tools also utilize simpler methods like bar charts. Unlike heatmaps, where explainability is incorporated within the text, other methods present explanations separately from the text and hence, more noticeable, making them more comprehensible. For instance, bars indicating the percentage of a criterion fulfillment are positioned alongside the image in resume screening platforms like ResyMatch.io. While some previous studies found that providing explanations can positively affect performance in a decision-making task^6,18^, others did not (e.g.,^32^). As findings on explainability are inconclusive, more research regarding this discrepancy is needed to investigate the role of explainable AI advice in decision-making and how explanations ought to be presented^33^. This need also extends to HRM, as a recent review of ways to enhance personnel selection processes advocated for more studies exploring the opacity of AI systems in different decision-making scenarios^34^. In line with most previous findings, we hypothesized that explainable advice positively affects decision-making in the personnel selection task.

To answer our research question on the source of advice and hypotheses on the accuracy and explainability of advice, we conducted multiple experiments using a personnel selection task to systematically investigate the effects of different advice feature characteristics. Consequently, we focused on how different *sources of advice* (AI vs. human), *accuracy* (correct vs. incorrect), and *explainability* (no explainability vs. two different explanation methods) *of advice* influence participants’ decision-making, using the dependent variables: a) task performance, b) advice quality rating, and c) individuals’ confidence in their decision.

## Methods

### General procedure across experiments

The preregistered (https://osf.io/dfwy6/) study was approved by the Research Ethics Committee of the University of Regensburg (identifier: 21-2475-101). All participants were informed about the purpose of the study and gave informed consent. Participation was voluntary and anonymity was ensured. The study was conducted in accordance with all relevant guidelines of the Research Ethics Committee of the University of Regensburg and the Ethical Principles of Psychologists and Code of Conduct outlined by the American Psychology Association (APA). Participants were recruited via university mailing lists and social media channels. Furthermore, we specifically targeted individuals with HRM experience via professional networking platforms such as LinkedIn and Xing. We conducted five online experiments. In each, participants had the role of a recruiter tasked with identifying suitable candidates for a specific position (Head of Quality Management at an automotive company). To achieve this, they had to review the two-page resumes of applicants against a list of selection criteria. All resumes used in the experiments were explicitly designed for this study so that precisely 50% of candidates met the selection criteria, and consequently, were suitable for the position. A detailed description of the design of the resumes and the selection criteria can be found in the supplementary material. Participants received two practice resumes with detailed feedback on whether their decisions regarding the candidates’ suitability were correct before moving on to the actual task. In each experiment, participants were presented with 16 resumes in a random order and then had to decide whether the applicant was suitable for the position. Participants were randomly assigned to one of the experimental groups or the control group. In the experimental groups, we presented the results of a purported prescreening of each resume, which participants could use as advice in making their decision. Generally, 75% of the advice was correct, and we randomized which resume was assigned to the correct or incorrect advice. The control group did not receive any additional information.

**Figure 1:**
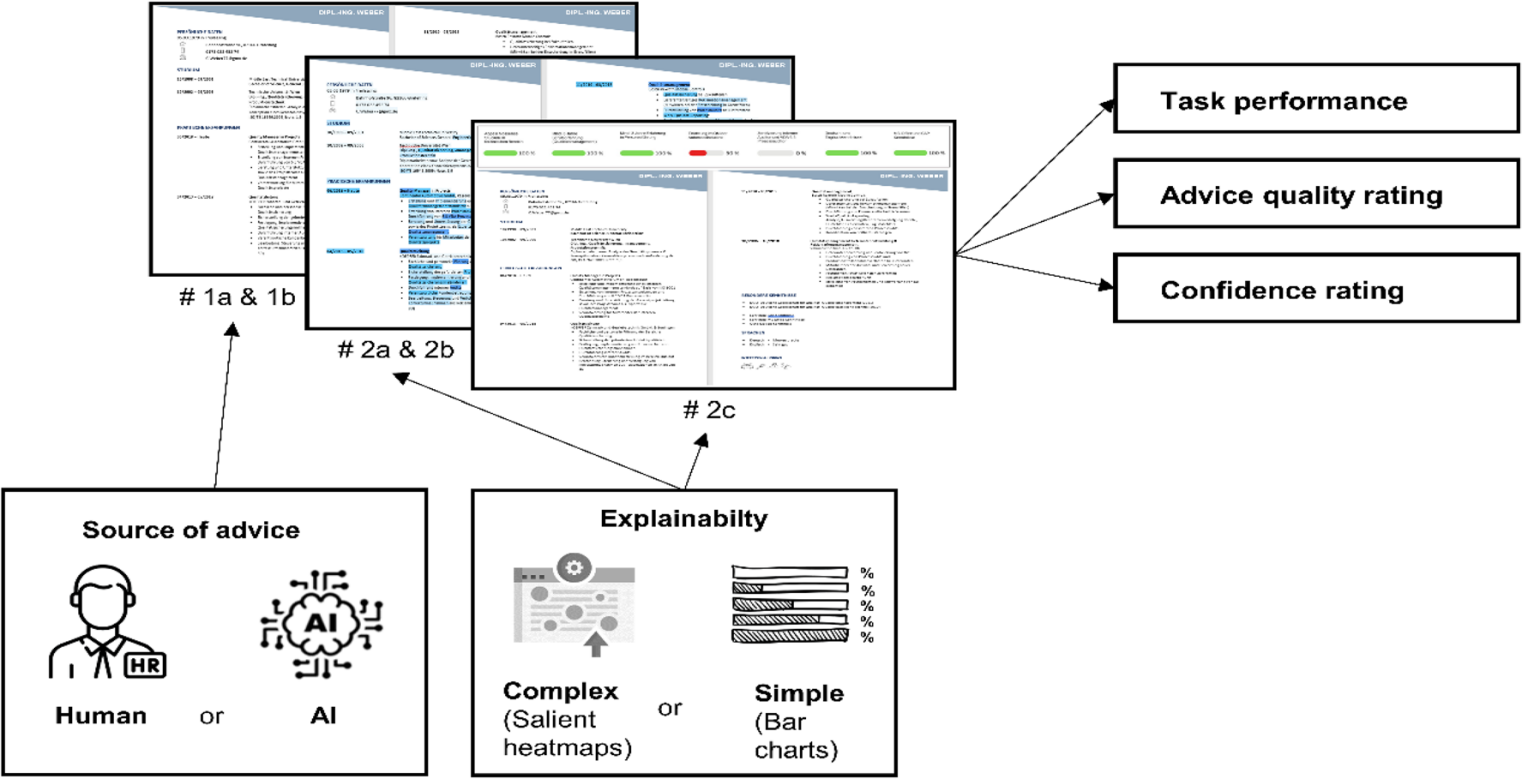
Experimental design. Each participant reviewed 16 CVs, with 50% of the CVs meeting the selection criteria. In Experiment 1a & b, they either received no advice or the advice was labeled as coming from a human or AI system. In Experiments 2a–c, the AI advice was either provided without or with explanations (heatmaps or charts). Participants had to rate the suitability of each CV, the quality of the advice (if applicable), and the confidence in their decision.

In addition, in the first two experiments (Experiment 1a, 1b), we manipulated the *source of advice* as coming from a human or an AI system to address our research question (testing for algorithmic aversion vs. appreciation). The advice’s *explainability method* was varied in the following three experiments (Experiment 2a, 2b, 2c) to test our hypothesis regarding the positive effect of explainable advice on decision-making. Table 1 provides an overview of the five experiments, including the wording used for each experimental condition. Experiment 1b was a replication of Experiment 1a, differing only in the sample composition (students vs. HRM employees). In Experiment 2b, we replicated Experiment 2a with only the allotted time to review the resumes being changed. Finally, Experiment 2c differed from Experiments 2a and 2b in the visualization technique used for the explanations. As Experiments 1b and 2b were replications of Experiments 1a and 2a, respectively, they were not preregistered separately. Besides having to rate each applicant’s suitability, the participants were asked to rate the perceived quality of the advice (only in the experimental groups) and the confidence in their decision (all groups). An overview of the experimental designs is outlined in Fig. 1.

**Table 1:** Manipulation wording of each experimental group across experiments.

*#*	Condition 1	Condition 2
1a & 1b	**Human advice** The resumes of the applicants were reviewed in a prescreening by your colleague, Julia Schmid. Ms. Schmid has recently joined the same company as you as a recruiter. For each resume, you will receive advice from the prescreening by Ms. Schmid	**AI advice** The resumes of the applicants were reviewed in a prescreening by the AI-based recruiting software AI-Hire. AI-Hire has recently been implemented in your company. For each resume, you will receive advice from the prescreening of AI-Hire
2a & 2b	**Non-Explainable AI Advice** The resumes of the applicants were reviewed in a prescreening by the AI-based recruiting software AI-Hire. For each resume, you will receive advice from the prescreening of AI-Hire	**Explainable AI Advice (Heatmap)** [see Condition 1] + AI-Hire works with automatic text recognition according to the principle of Natural Language Processing (NLP), in which texts are analyzed with the help of complex algorithms. Heatmaps are often used for this purpose. Heatmaps are used to visually highlight those parts of the text that appear to be particularly relevant to the system’s task. AI-Hire works with color coding of single text modules or words. The following scale shows the color gradations used by AI-Hire: the darker the color, the more important the word is from the perspective of AI-Hire [Heatmap presented]
2c	**Non-Explainable AI Advice** The resumes of the applicants were reviewed in a prescreening by the AI-based recruiting software AI-Hire. For each resume, you will receive advice from the prescreening of AI-Hire	**Explainable AI advice (Charts)** [see Condition 1] + AI-Hire works with automatic text recognition according to the principle of Natural Language Processing (NLP), in which texts are analyzed with the help of complex algorithms. The description of the result of the screening is visualized for each criterion depending on the degree of suitability on a scale of 0–100%. Suitability only exists if the criterion is fully met (100%)

### Dependent variables

In all experiments, the three dependent variables were based on established measures^17,18^.

#### Performance

After reviewing each resume, participants were asked to rate whether the applicant was suitable for the advertised position by selecting “suitable” or “unsuitable”. Each decision was scored as 0 (incorrect) and 1 (correct). Participants’ performance was calculated as the percentage of correct decisions.

#### Advice quality rating

In conditions with advice, its quality was measured as the mean of two items: Participants rated the perceived usefulness (“How useful was the prescreening advice for your decision?”) and trust (“How much do you trust [source of advice]?”) on a 7-point Likert scale from 1 (*not at all)* to 7 (*extremely*). Advice quality ratings showed a very good internal consistency (Cronbach’s α ≥ 0.86 (0.86–0.97)).

#### Confidence rating

Participants’ confidence in their decision was assessed with one item (“How confident are you in your decision?”) on a 7-point Likert scale from 1 (*not at all*) to 7 (*extremely*).

### Statistical analysis

The data analysis was performed using R (Version 4.1.2). Participants were excluded from the data analysis if they did not finish the survey, failed a manipulation or attention check item, or completed the study in an unrealistic short time. A power analysis was conducted for Experiments 1a and 1b, resulting in a minimum sample size of 60 participants per experimental condition to have a power of at least 0.80, using effect estimates from a related study^17^.

In all experiments, we compared the overall task performance between the control and experimental groups using one-way ANOVAs and post-hoc Tukey-HSD for differences between groups. Three mixed-effects regressions were calculated for each experiment, one per dependent variable. Logistic regression was used for the performance analyses as the performance was assessed as a binary variable (correct vs. incorrect). Linear regressions were used to analyze the advice quality and confidence ratings. All three models were regressed on the advice’s accuracy, the source of advice or explainability of advice, and the interaction between accuracy and source/explainability. We controlled for potentially relevant variables (attitude towards AI, need for autonomy, need for competence, affinity for AI, AI knowledge, and professional experience). See supplementary material for a detailed description of the control variables, including the reason for their inclusion, the respective internal consistency of the scales, and all models controlled for covariates. The independent variables were included in each model as fixed effects. Participant ID and resume number were included as random effects. The participant ID was included to account for individual differences and the non-independence of the observations. The individual resumes represented the second random effect accounting for different (CV) design types and difficulty levels. The experiments were conducted in German. Translated versions of the materials into English can be made available upon request. The German material, data, analysis scripts, and preregistrations for all experiments are publicly accessible at https://osf.io/dfwy6/.

## Experiment 1a: Accuracy and source of advice

### Method

#### Participants and design

The sample in Experiment 1a consisted of *N* = 370 university students (80.3% female, 19.5% male, 0.3% diverse, *M*_age_ = 25.31, *SD*_age_ = 5.48) with 30.3% working part-time and 50.5% having some work experience in HRM. Their average HRM work experience in years was *M* = 1.89 (*SD* = 1.85). Participants could review each resume for max. 45 s, as the average review time for resumes in real life is less than 1 min^35^. Individuals were allocated into control (no advice) and experimental (advice) groups. The experiment followed a 2 × 2 mixed-factors design with *source of advice* (AI vs. human) as the between-subject factor and *accuracy of advice* (correct vs. incorrect) as the within-subject factor.

#### Results

ANOVA: First, we tested whether the presentation of advice impacted the performance. Overall performance did not significantly differ when participants received advice in comparison to no advice, *F*(2, 367) = 1.24, *p* = 0.290, η_p_^2^ = 0.01. Mean performance rates over all experiments and conditions can be found in Table 2.

**Table 2:** Mean performance rates over experiments for different types of advice.

	No advice	Human	AI: Non-Explainable	AI: Explainable	Correct	Incorrect
Experiment 1a	68.17 (46.59)	70.41 (45.66)	67.40 (46.89)	–	72.74 (44.57)	57.60 (49.44)
Experiment 1b	68.20 (46.59)	66.96 (47.05)	68.11 (46.62)	–	71.40 (45.20)	55.86 (49.69)
Experiment 2a	70.61 (45.57)	–	73.70 (44.04)	74.42 (43.64)	78.39 (41.17)	61.10 (48.78)
Experiment 2b	72.67 (44.60)	–	72.77 (44.55)	73.59 (44.11)	76.93 (42.15)	62.11 (48.58)
Experiment 2c	72.04 (44.89)	–	72.61 (44.61)	74.30 (43.71)	76.88 (42.17)	62.98 (48.32)

Numbers in brackets indicate standard deviations.

**Figure 2:**
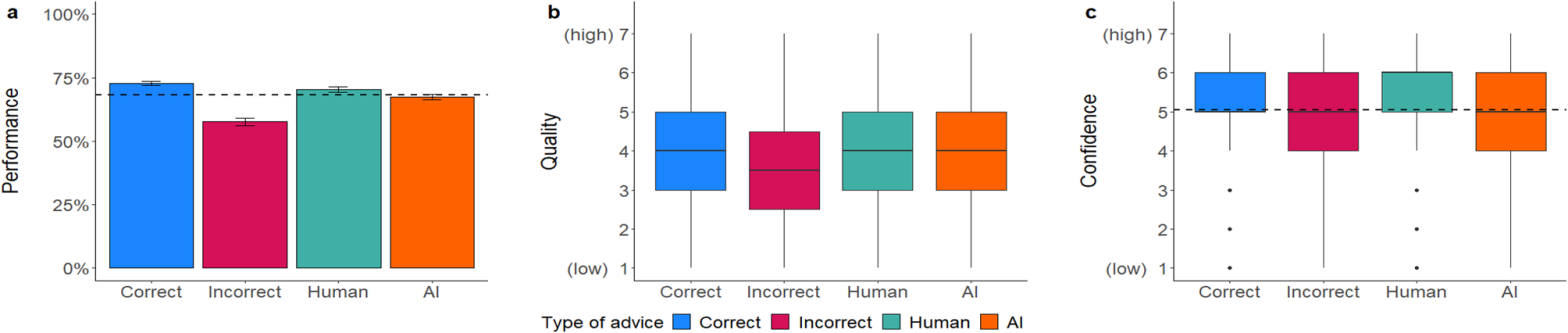
Dependent variables across manipulations in Experiment 1a. Figure (**a**) shows the performance in percentage, (**b**) the quality ratings, and (**c**) the confidence ratings. Error bars indicate standard errors. The boxplots show the 25th to 75th percentiles with the median as the central line. The whiskers extend to a maximum of 1.5 × interquartile range. The dotted line represents the average baseline performance (*M* = 68.17, *SD* = 46.59) and confidence ratings (*M *= 5.05, *SD* = 1.47).

Mixed regression models: Participants’ performance was significantly better when they received correct (vs. incorrect) advice and when the advice was labeled as coming from the human (vs. AI; see Fig. 2a). However, the latter effect was no longer significant when controlling for the covariates (see Table S1 in the supplementary material). Similarly, we found that the advice’s quality rating was significantly higher when receiving correct advice, while the effect of source of advice on the advice quality rating was non-significant (see Fig. 2b). Further, there were significant effects of accuracy and source of advice on participants’ confidence in their own decision, with confidence being higher when advice was correct and came from the human (see Fig. 2c). When covariates were controlled for, the effect of accuracy on confidence was no longer statistically significant. We did not find any significant interaction effects between accuracy and source of advice.

**Figure 3:**
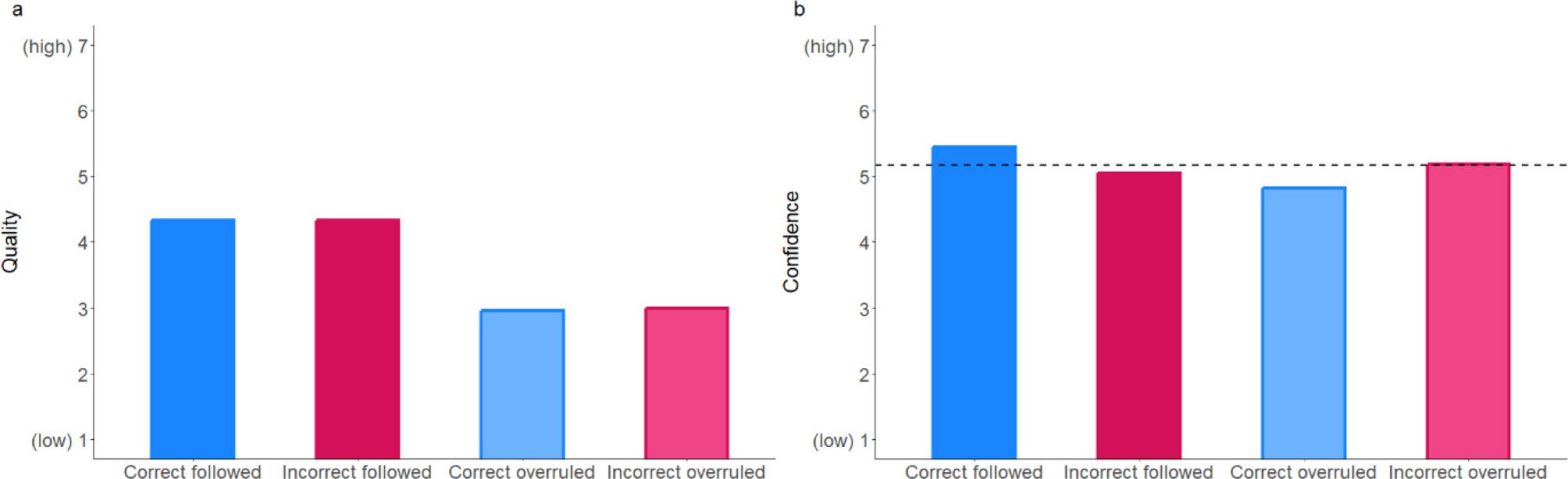
Quality ratings (**a**) and confidence ratings (**b**) across the different combinations of advice and participants’ decision. Correct followed = Correct advice and participants’ decision was correct, Incorrect followed = Incorrect advice and participants’ decision was incorrect, Correct overruled = Correct advice but participants’ decision was incorrect, Incorrect overruled = Incorrect advice but participants’ decision was correct.

We further examined the quality and confidence ratings across the different combinations of advice and participant decisions (see Fig. 3). Results show that the quality of the advice was rated similarly in both conditions where participants followed the advice, even if it was incorrect. Additionally, when correct advice was followed, confidence was significantly higher than at baseline (*t*(4023) = 9.50, *p* < 0.001) and dropped to or below the baseline when the advice was overruled or incorrectly followed, *t*(1633.6) = -4.12, *p* < 0.001 for correct overruled; *t*(1000.2) = 2.10, *p* = 0.036 for incorrect overruled. Across all experiments, quality and confidence ratings were similar (see Table S16).

Our results show that the advice’s accuracy strongly influenced our participants’ performance, which improved when receiving correct advice, while people often also failed to dismiss incorrect advice, leading to lower performance and overreliance on incorrect advice, supporting our hypothesis on accuracy (see Table 3). Nevertheless, respondents rated the quality of incorrect advice lower than correct advice and incorrect advice led to lowered confidence ratings. This indicates that participants were able to detect incorrect advice in some cases. However, participants who followed incorrect advice did not understand that the advice was incorrect, as the quality was rated similarly high to the correct advice followed and the confidence was still higher than in the correct overruled condition (see Fig. 3).

**Table 3:** Mixed multilevel regressions for performance, advice quality, and confidence ratings in Experiment 1a.

Predictors	Performance				Quality				Confidence			
	OR	SE	z	*p*	β	SE	t	*p*	β	SE	t	*p*
Intercept	1.21	0.23	1.02	.307	3.52	0.11	32.55	** < .001**	4.99	0.09	53.86	** < .001**
Accuracy [Correct]	2.33	0.27	7.31	** < .001**	0.37	0.06	6.71	** < .001**	0.18	0.06	3.27	** < .001**
Source [Human]	1.35	0.19	2.51	**.031**	0.07	0.15	0.50	.617	0.26	0.11	2.30	**.022**
Accuracy x Source	0.81	0.13	-1.30	.195	0.05	0.08	0.59	.557	-0.06	0.08	-0.77	.442
Random effects												
σ^2^	3.29				1.06				1.08			
τ_00_	0.06 _ID_				1.06 _ID_				0.47 _ID_			
	0.41 _CV_				0.00 _CV_				0.03 _CV_			
ICC	0.12				0.50				0.32			
Marginal R^2^	0.03				0.02				0.01			
Conditional R^2^	0.15				0.51				0.33			

*N*_ID_ = 240, *N*_CV_ = 16, Observations = 3840.

OR > 1 variable associated with higher odds for correct decision; OR < 1 variable associated with lower odds for correct decision; OR = 1 variable does not affect the outcome odds.

The regression estimate *β* indicates how much the mean quality rating changes given a one-unit shift in the predictor while holding other predictors in the model constant. Bold values indicate significant effects.

Regarding the source of advice, results showed a tendency toward algorithmic aversion as people seemed to benefit less from AI advice. However, the effect was small and diminished when we controlled for covariates. Due to the combination of slightly improved performance and higher confidence ratings when receiving human advice, we assume that participants were more comfortable following human advice.

## Experiment 1b: Replication of 1a with HRM employees

### Method

The sample of Experiment 1a consisted only of students with no or little expertise in HRM. Consequently, we replicated this experiment with more experienced HRM employees to see if any of the results would be affected by the level of experience in the targeted group.

#### Participants and design

Participants in Experiment 1b were employees in HRM (*N* = 242, 71.1% female, 28.9% male, *M*_age_ = 26.93, *SD*_age_ = 5.55). Most of the participants were working in selection/recruiting (54.5%), followed by administration (23.6%) and development (13.6%), and others (8.3%) with a mean HRM work experience in years of *M* = 3.13 (*SD* = 4.15). Experiment 1b followed the same basic design as Experiment 1a.

### Results

ANOVA: When comparing the performance rates between the control and both experimental groups, we found no statistically significant difference, *F*(2, 239) = 0.32, *p* = 0.730, η_p_^2^ = 0.00 (see Table 3).

**Figure 4:**
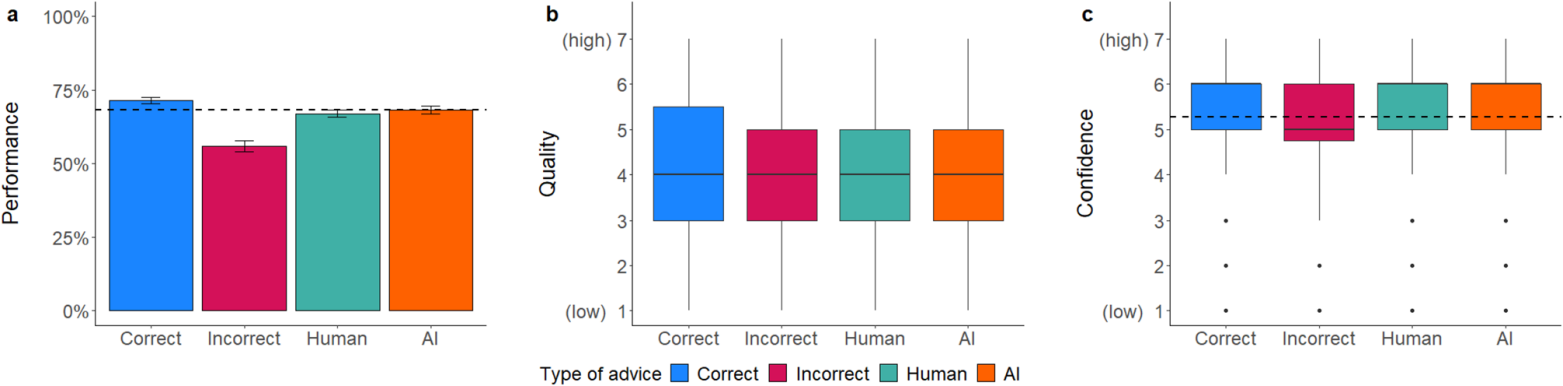
*Dependent variables across manipulations in Experiment 1b*. Figure (**a**) shows the performance rates, (**b**) the quality ratings, and (**c**) the confidence ratings. Error bars indicate standard errors. The boxplots show the 25th to 75th percentiles with the median as the central line. The whiskers extend to a maximum of 1.5 × interquartile range. The dotted line represents the average baseline performance (*M* = 68.20, *SD* = 46.59) and confidence ratings (*M* = 5.27, *SD* = 1.36).

Mixed regression models: As expected, the accuracy of advice had a significant influence on all three dependent variables (Table 4). Performance, perceived advice quality, and confidence in their decisions were significantly higher when receiving correct vs. incorrect advice (Fig. 4), replicating the accuracy findings of the previous study among task novices in the more experienced group of HRM employees and supporting our hypothesis on accuracy. They often followed incorrect advice, even though they judged incorrect advice on average to be of lower quality than correct advice. We did not find a statistically significant effect for both, source of advice and the interaction between accuracy and source, on any of the dependent variables. The effects remained stable after controlling for covariates.

**Table 4:** Mixed multilevel regressions for performance, advice quality, and confidence ratings in Experiment 1b.

Predictors	Performance				Quality				Confidence			
	OR	SE	z	*p*	β	SE	t	*p*	β	SE	t	*p*
Intercept	1.22	0.25	0.98	.328	3.90	0.13	29.63	** < .001**	5.26	0.10	53.99	** < .001**
Accuracy [Correct]	2.41	0.34	6.25	** < .001**	0.41	0.07	5.91	** < .001**	0.18	0.07	2.63	** < .001**
Source [Human]	1.14	0.19	0.79	.427	− 0.25	0.18	− 1.40	.163	− 0.06	0.13	− 0.50	.616
Accuracy × Source	0.76	0.15	− 1.39	.164	0.02	0.10	0.18	.859	0.09	0.09	0.93	.353
Random effects												
σ^2^	3.29				1.10				1.04			
τ_00_	0.00 _ID_				1.07 _ID_				0.38 _ID_			
	0.43 _CV_				0.00 _CV_				0.02 _CV_			
ICC	0.11				0.49				0.28			
Marginal R^2^	0.03				0.02				0.01			
Conditional R^2^	0.14				0.50				0.280			

*N*_ID_ = 162, *N*_CV_ = 16, Observations = 2592.

OR > 1 variable associated with higher odds for correct decision; OR < 1 variable associated with lower odds for correct decision; OR = 1 variable does not affect the outcome odds.

The regression estimate *β* indicates how much the mean quality rating changes given a one-unit shift in the predictor while holding other predictors in the model constant. Bold values indicate significant effects.

**Figure 5:**
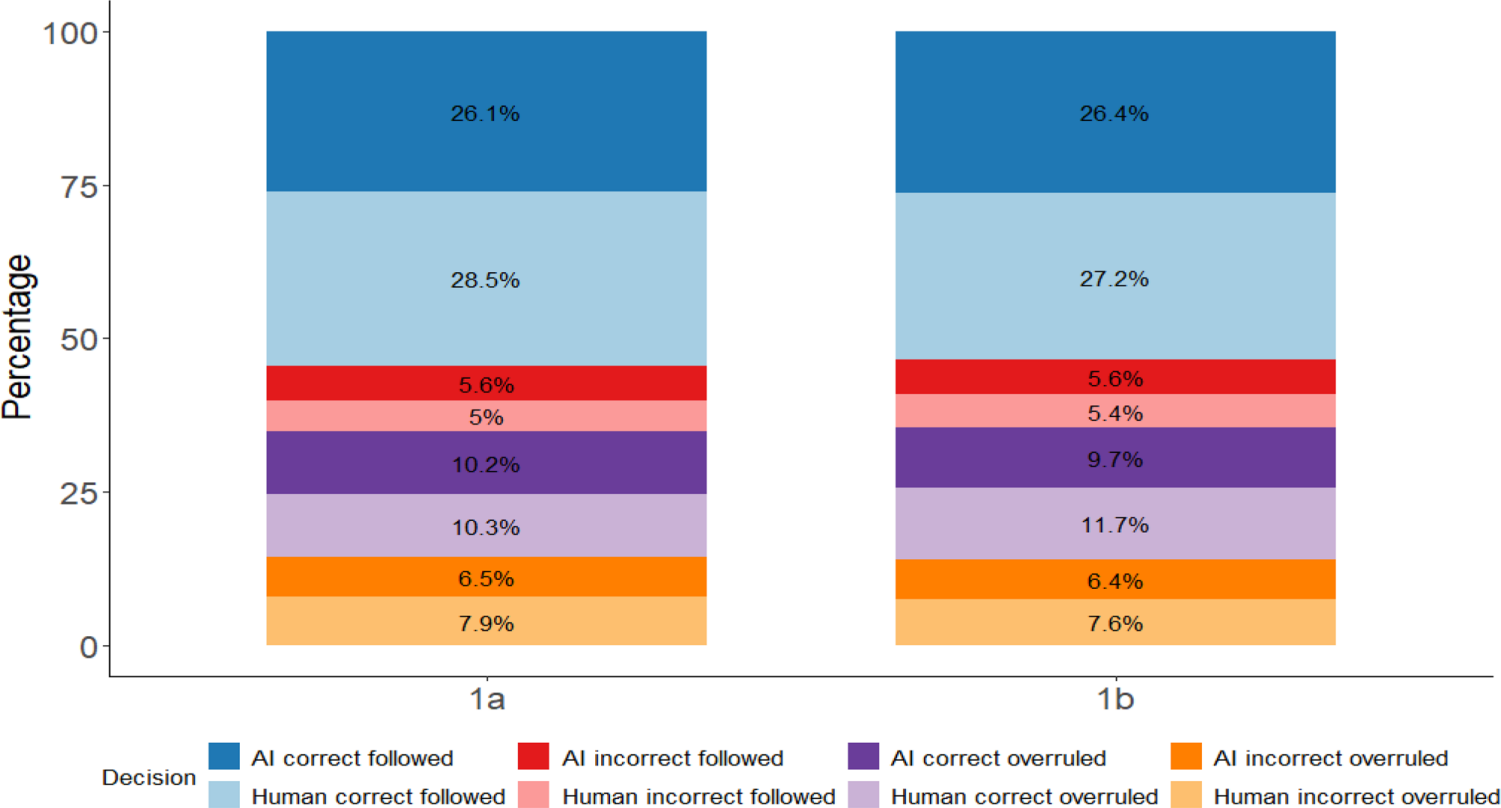
*Percentage overview of the different combinations of type of advice and participant decisions for Experiment 1a and 1b*. Correct followed = Presentation of correct advice and participants’ decision was correct, Incorrect followed = Presentation of incorrect advice and participants’ decision was incorrect, Correct overruled = Presentation of correct advice but participants’ decision was incorrect, Incorrect overruled = Presentation of incorrect advice but participants’ decision was correct.

Further, we explored the percentages of participants following or overruling correct or incorrect advice for Experiment 1a and 1b, displayed in Fig. 5. Experts showed similar rates of following correct and incorrect advice and overruling incorrect advice, whether it was human or AI advice. These findings indicate that more experienced people overall seemed to show less discrimination between the two sources of advice compared to people with less experience and showed no tendency towards algorithm aversion.

## Experiment 2a: Accuracy and explainability of advice

### Method

Since performance did not differ greatly between Experiment 1a and 1b, we concluded that novices and more experienced people show similar patterns in decision-making in this personnel selection task. Consequently, we conducted the following experiments (Experiments 2a, 2b, 2c) with full-time students and part-time working students. Since the source of advice had relatively little impact on decision-making, and we neither found a strong trend for algorithm aversion nor appreciation, we focused on testing whether explainability could help to prevent following incorrect advice, reducing the overreliance on AI advice.

#### Participants and design

The sample included *N* = 328 students (77.1% female, 22% male, 0.3% diverse, 0.6% preferred not to answer, *M*_age_ = 25.47, *SD*_age_ = 4.96) with 42.7% reporting experience in HRM with a mean of *M* = 1.79 years (*SD* = 1.97). Participants in Experiments 1a and 1b mentioned that the screening time of 45 s was too limited. Therefore, we expanded the screening time to 60 s for this experiment. As before, participants were randomly assigned to one of three groups: (a) no advice, (b) AI advice without explanation, or (c) AI advice with a visual explanation. Research suggests aligning the choice of an explainability method with the specific objectives the explanations aim to achieve^36^. In our study, the main goal was to facilitate the identification of incorrect advice for users and thus reduce overreliance. Given that the CVs consist of textual data, we chose saliency heatmaps as a visual explanation method. By utilizing different color shades to emphasize the varying importance of text features^27–31^, users can quickly identify the key phrases influencing the applicant’s suitability and the AI’s decision. This might help them understand the rationale behind the AI advice and evaluate its validity more effectively, facilitating the identification of incorrect advice. Accordingly, words or phrases on the resumes were color-coded depending on their perceived relevance according to the AI prediction. To examine the effect of accuracy and explainability of the advice on the dependent variables, a 2 × 2 mixed factorial design was used. As before, *accuracy of advice* (correct vs. incorrect) was included as the within-subject factor. *Explainability of advice* (explainable advice vs. non-explainable advice) represented the between-subject factor. Dependent variables were the same as in Experiments 1a and 1b.

#### Results

ANOVA: Looking at the overall performance rates, we found a statistically significant difference between the control group without and experimental groups with advice, *F*(2, 325) = 3.25; *p* = 0.040, η_p_^2^ = 0.02. Out of all three groups, only performance in the condition with explainable advice was significantly higher compared to receiving no advice (see Table 3).

**Figure 6:**
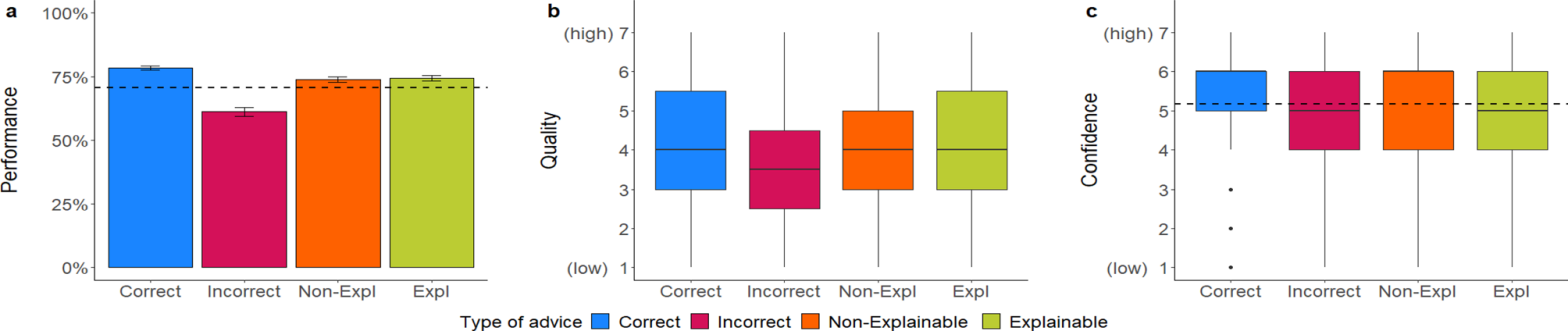
*Dependent variables across manipulations in Experiment 2a*. Figure (**a**) shows the performance rates, (**b**) the quality ratings, and (**c**) the confidence ratings. Error bars indicate standard errors. The boxplots show the 25th to 75th percentiles with the median as the central line. The whiskers extend to a maximum of 1.5 × interquartile range. The dotted line represents the average baseline performance (*M* = 70.61, *SD* = 45.57) and confidence ratings (*M* = 5.17, *SD* = 1.38).

Mixed regression models: As shown in Table 5, participants performed significantly better (Fig. 6a), rated the quality of the advice higher (Fig. 6b), and showed more confidence in their own decisions when receiving correct advice (Fig. 6c). Again, this supports our hypothesis on accuracy. The similar effects for advice accuracy as in the previous experiments highlight that the quality of the AI system had a consistently strong impact on participants’ performance in our personnel selection task.

**Table 5:** Mixed multilevel regressions for performance, advice quality and confidence ratings in Experiment 2a.

	Performance				Quality				Confidence			
Predictors	OR	SE	z	*p*	β	SE	t	*p*	β	SE	t	*p*
Intercept	1.46	0.27	2.02	**.044**	3.54	0.12	30.12	** < .001**	5.08	0.11	46.26	** < .001**
Accuracy [Correct]	2.85	0.36	8.33	** < .001**	0.59	0.06	9.98	** < .001**	0.24	0.06	3.71	** < .001**
Explainability [Explainable]	1.26	0.19	1.57	.116	0.24	0.16	1.45	.146	-0.06	0.14	-0.41	.681
Accuracy × Explainability	0.76	0.13	-1.56	.119	0.01	0.08	0.12	.903	0.13	0.09	1.40	.161
Random effects												
σ^2^	3.29				1.12				1.28			
τ_00_	0.05 _ID_				1.15 _ID_				0.67 _ID_			
	0.39 _CV_				0.01 _CV_				0.04 _CV_			
ICC	0.12				0.51				0.36			
Marginal R^2^	0.04				0.04				0.01			
Conditional R^2^	0.15				0.53				0.37			

*N*_ID_ = 214, *N*_CV_ = 16, Observations = 3424.

OR > 1 variable associated with higher odds for correct decision; OR < 1 variable associated with lower odds for correct decision; OR = 1 variable does not affect the outcome odds.

The regression estimate *β* indicates how much the mean quality rating changes given a one-unit shift in the predictor while holding other predictors in the model constant. Bold values indicate significant effects.

Against our hypothesis, no significant effect for explainable advice or the interaction between accuracy and explainability was found for any of the dependent variables. When we controlled for covariates, all effects remained stable. Providing AI advice with a visual explanation for its prediction by using a widely employed approach (i.e., salient heatmaps) apparently did not facilitate recognition of incorrect advice.

## Experiment 2b: Replication of 2a without time limit

### Method

As salient heatmaps are a relatively advanced method of explaining AI predictions, some participants found their complexity cognitively overwhelming. Additionally, even with the resume viewing time of 60 s in Experiment 2a, many participants still mentioned time pressure when screening the resumes. Hence, we conducted the same experiment as in Experiment 2a with unlimited reviewing time to determine whether time pressure might explain why the participants did not benefit from explainable advice.

#### Participants and design

The sample in Experiment 2b consisted of *N* = 143 students (69.9% female, 30.1% male, *M*_age_ = 26.96, *SD*_age_ = 6.17) and 47.6% had prior experience in HRM, with a mean of *M* = 1.92 years (*SD* = 2.93). The experiment was an exact replication of Experiment 2a.

#### Results

ANOVA: We found no statistically significant difference in the overall performance rates between the control group without advice and the two experimental groups with advice, *F*(2, 140) = 0.09, *p* = 0.913, η_p_^2^ = 0.00.

**Figure 7:**
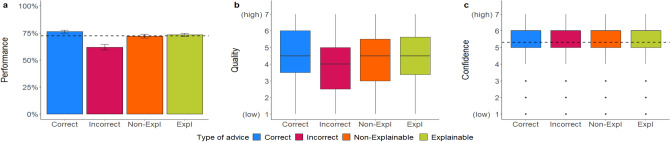
*Dependent variables across manipulations in Experiment 2b*. Figure (**a**) shows the performance rates, (**b**) portrays quality ratings, and (**c**) confidence ratings. Error bars indicate standard errors. The boxplots show the 25th to 75th percentiles with the median as the central line. The whiskers extend to a maximum of 1.5 × interquartile range. The dotted line represents the average baseline performance (*M* = 72.66, *SD* = 44.60) and confidence ratings (*M* = 5.38, *SD* = 1.24).

Mixed regression models: As seen in Table 6 and in line with our hypothesis, the accuracy of advice significantly affected participants’ performance, advice quality rating, and confidence in their own decision, similar to Experiment 2a. Even without a time limit to review the resumes and contrary to our hypothesis, the explainability of the advice did not affect participants’ performance nor their confidence, suggesting that even with infinite time to review the resumes and AI advice, visualized explanations for why the AI made its prediction evidently did not help participants recognize when the AI was incorrect. However, receiving explainable advice led to slightly more favorable advice quality ratings (Fig. 7), but this effect was no longer statistically significant when covariates were controlled for.

**Table 6:** Mixed multilevel regressions for performance, advice quality, and confidence ratings in Experiment 2b.

Predictors	Performance				Quality				Confidence			
	OR	SE	Z	*p*	β	SE	t	*p*	β	SE	t	*p*
Intercept	2.00	0.46	3.02	**.003**	3.52	0.19	18.63	** < .001**	5.44	0.13	41.30	** < .001**
Accuracy [Correct]	1.68	0.34	2.57	**.010**	0.81	0.10	8.30	** < .001**	0.16	0.08	2.00	**.045**
Explainability [Explainable]	0.75	0.17	− 1.25	.213	0.50	0.25	1.98	**.048**	0.20	0.17	1.14	.254
Accuracy × Explainability	1.62	0.43	1.81	.070	− 0.25	0.13	− 1.94	.052	− 0.18	0.11	− 1.67	.095
Random effects												
σ^2^	3.29				1.20				0.85			
τ_00_	0.09 _ID_				1.19 _ID_				0.48 _ID_			
	0.34 _CV_				0.00 _CV_				0.01 _CV_			
ICC	0.12				0.50				0.37			
Marginal R^2^	0.03				0.04				0.00			
Conditional R^2^	0.15				0.52				0.37			

*N*_ID_ = 95, *N*_CV_ = 16, Observations = 1520.

OR > 1 variable associated with higher odds for correct decision; OR < 1 variable associated with lower odds for correct decision; OR = 1 variable does not affect the outcome odds.

The regression estimate *β* indicates how much the mean quality rating changes given a one-unit shift in the predictor while holding other predictors in the model constant. Bold values indicate significant effects.

## Experiment 2c: Accuracy and explainability (simplified) of advice

### Method

As can be deduced from the results of Experiments 2a and 2b, it is feasible that saliency heatmaps are simply not an optimal method for explaining the underlying process of the AI prediction to end users. Given our goal of aiding users in detecting incorrect advice, in Experiment 2c, we utilized a different explanation method based on a more user-friendly technique. Specifically, we displayed the applicants’ degree of suitability for the position for each screened criterion. Each criterion was visualized through a bar, with bars displayed in red if the criterion was not fully met (less than 100%) and in green if fully met. This visualization method enables users to quickly identify disparities between the AI advice and the actual suitability of an applicant. For instance, if the visualization shows all green bars for every criterion, implying that all are met, but the applicant is still rated as not suitable by the AI, it becomes more evident that the advice is likely incorrect. This was expected to enhance the understanding of the criteria on which the AI based its decision and thereby aiding in the identification of incorrect AI advice. This method of explaining the decisions of AI systems is already used by available systems in the field of personnel selection (e.g., ResyMatch.io).

#### Participants and design

In Experiment 2c, the sample consisted of *N* = 320 full-time and part-time working students (72.8% female, 25.3% male, 0.3% diverse, 1.6% preferred not to answer, *M*_age_ = 24.02, *SD*_age_ = 6.14) with 25.3% having experience in HRM and a mean of *M* = 2.36 years (*SD* = 4.50). The experiment followed the same design as Experiments 2a and 2b, but the visualization method in the explainability condition differed. Explainable advice was visualized for each criterion depending on the degree of suitability on a scale of 0–100% using bar graphs. Data were analyzed identically to Experiments 2a and 2b.

#### Results

ANOVA: Similar to the previous experiments, we did not find a significant difference in the performance rates between the control group and the two experimental groups, *F*(2, 317) = 1.15; *p* = 0.347, η_p_^2^ = 0.01.

**Figure 8:**
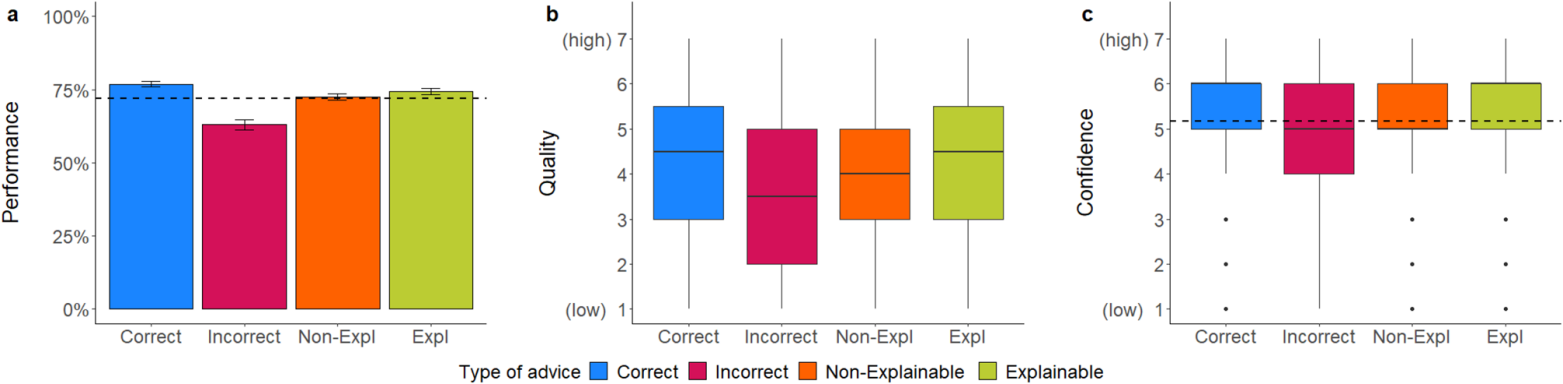
*Dependent variables across manipulations in Experiment 2c*. Figure (**a**) shows the performance rates, (**b**) portrays quality ratings, and (**c**) confidence ratings. Error bars indicate standard errors. The boxplots show the 25th to 75th percentiles with the median as the central line. The whiskers extend to a maximum of 1.5 × interquartile range. The dotted line represents the average baseline performance (*M* = 72.04, *SD* = 44.89) and confidence ratings (*M* = 5.18, *SD* = 1.43).

Mixed regression models: As expected and shown in Table 7, the presentation of correct advice significantly increased participants’ performance, perceived advice quality and confidence rating, and vice versa (see Fig. 8). Consistent with all previous experiments and our hypothesis, these results indicate that participants trusted their advice and own decisions less when receiving incorrect advice but still often failed to dismiss it, showing overreliance on incorrect advice. Providing explainable advice positively affected the respondents’ confidence in their decision, but no significant effects on performance and advice quality ratings were found. This indicates that providing explanations for the AI prediction did not facilitate the recognition of erroneous advice.

**Table 7:** Mixed multilevel regressions for performance, advice quality, and confidence ratings in Experiment 2c.

Predictors	Performance				Quality				Confidence			
	OR	SE	Z	*p*	β	SE	t	*p*	β	SE	t	*p*
Intercept	1.74	0.34	2.83	**.005**	3.57	0.12	29.50	** < .001**	4.97	0.10	48.07	** < .001**
Accuracy [Correct]	2.07	0.26	5.91	** < .001**	0.60	0.06	9.61	** < .001**	0.43	0.06	7.57	** < .001**
Explainability [Explainable]	1.07	0.17	0.41	.683	− 0.01	0.17	− 0.07	0.946	0.34	0.13	2.65	**.008**
Accuracy × Explainability	1.05	0.19	0.26	.793	0.24	0.09	2.61	**.009**	− 0.38	0.08	− 4.53	** < .001**
Random effects												
σ^2^	3.29				1.29				1.07			
τ_00_	0.08 _ID_				1.22 _ID_				0.57 _ID_			
	0.43 _CV_				0.01 _CV_				0.05 _CV_			
ICC	0.13				0.49				0.37			
Marginal R^2^	0.03				0.04				0.01			
Conditional R^2^	0.16				0.51				0.37			

*N*_ID_ = 208, *N*_CV_ = 16, Observations = 3328.

OR > 1 variable associated with higher odds for correct decision; OR < 1 variable associated with lower odds for correct decision; OR = 1 variable does not affect the outcome odds.

The regression estimate *β* indicates how much the mean quality rating changes given a one-unit shift in the predictor while holding other predictors in the model constant. Bold values indicate significant effects.

**Figure 9:**
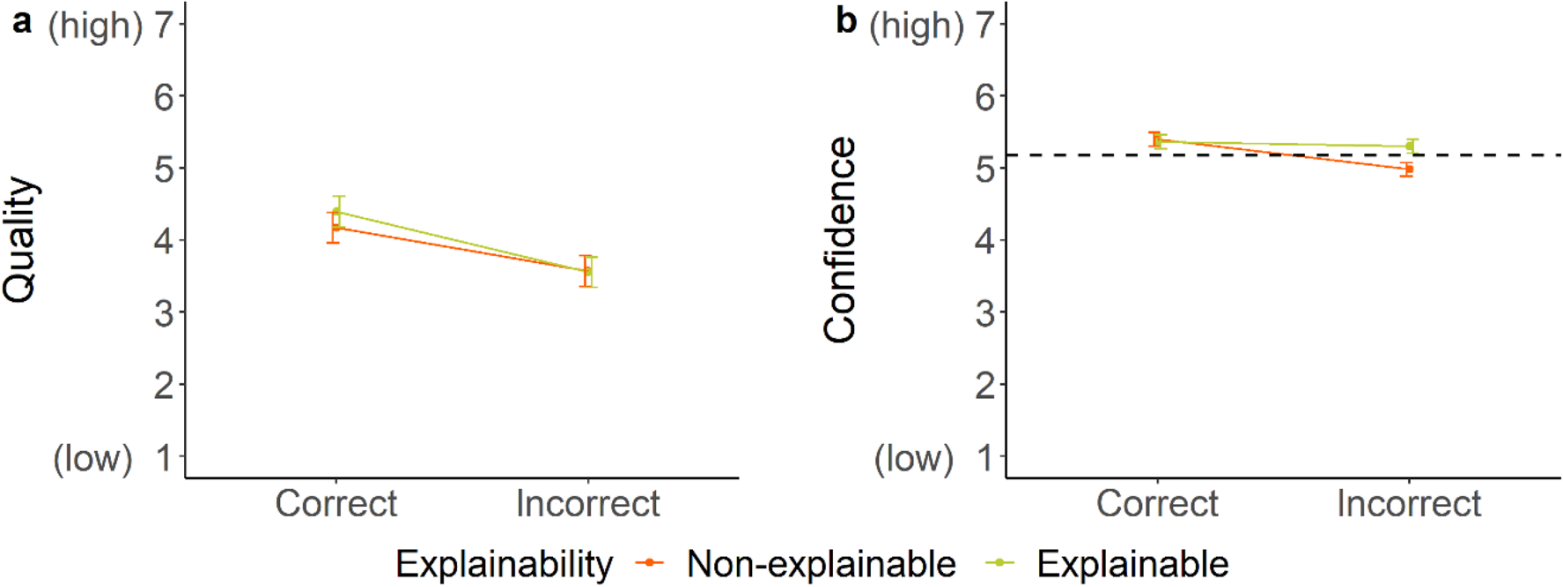
*Mean quality* (**a**) *and confidence ratings* (**b**) *as a function of accuracy and explainability of advice.* The dotted line represents the average baseline confidence ratings for the control group (*M* = 5.18, *SD* = 1.43).

For the first time, we found a significant interaction between advice accuracy and explainability for both, the advice quality and confidence ratings. In line with our hypothesis, the quality of correct advice was rated higher when receiving explainable AI advice, while quality ratings were not affected by explainability when receiving incorrect advice (see Fig. 9a). Surprisingly, participants expressed more confidence in their decision when receiving explanations for incorrect advice compared to the baseline with no advice; but, when receiving correct advice, explainability did not affect their confidence (see Fig. 9b). Further, participants were less confident after receiving non-explainable incorrect advice than when they received no advice at all. All effects remained stable after controlling for covariates.

## Discussion

Our aim was to investigate AI-generated advice-taking in personnel decision-making. Providing advice had a strong impact on our participants’ behavior and different advice characteristics affected their decisions to varying degrees.

First, looking at the *source of advice*, we found that novices performed slightly better when receiving human advice. When comparing novices and experts, we additionally observed that the difference between the percentage of participants who followed correct human advice and those who followed correct AI advice was greater among novices. However, this tendency towards algorithmic aversion disappeared when controlling for covariates and did not show among more experienced participants. Overall, our results show little evidence for algorithmic appreciation or aversion. These findings are somewhat inconsistent with other research, indicating that people with less task expertise might show algorithmic appreciation^15^, or that task experts show algorithmic aversion^17^. However, the findings contribute to research indicating that algorithmic aversion is task-dependent, with reduced reliance on algorithms in more subjective tasks^37^. While personnel selection should ideally be objective, the inevitable subjective tendencies in the process may have contributed to the observed results.

Second, the *accuracy of advice* played a pivotal role throughout all experiments. When receiving incorrect advice, participants’ performance consistently dropped below the level shown by individuals who did not receive advice. When receiving correct advice participants’ performance slightly improved compared to baseline levels, supporting our hypothesis. Participants who followed the incorrect advice rated quality similar to the correct advice. This indicates that they did not realize that the advice was incorrect, resulting in overreliance. In turn, participants who overruled the incorrect advice rated its quality lower, having realized its inaccuracy and actively decided to disregard it. Even though the overall advice quality and participants’ confidence were rated lower when receiving incorrect advice throughout all experiments (in line with previous findings^38^), participants still failed to dismiss incorrect advice, ultimately relying on it. Across all experiments, participants followed correct and incorrect advice in over two-thirds of their decisions. Correct advice was followed in approximately half of their decisions and incorrect advice in about a tenth. Other studies have found similar effects^17,19^, indicating that people often follow advice regardless of whether it is correct or not^9,10,16^. Participants’ tendency towards overreliance may be attributed to the advice serving as a decision anchor. In such instances, participants accept the AI advice without considering contradictory information, as it directs their attention towards aspects consistent with the advice^12^. Research has shown that adjusting one’s decision based on the anchor, in this case, the advice, occurs independently of prior judgements regarding the advice and is unintentional^10^. This independent information processing might explain people’s reliance on incorrect advice, despite rating its overall quality lower and showing less confidence in their decisions.

Third, we tested whether *explainability* positively affects decision-making, and therefore, could reduce overreliance on incorrect advice by describing how the AI system makes its prediction. Against our hypothesis, neither explainability manipulation led to a significant reduction in overreliance on incorrect advice, nor did it improve overall performance. Contrary to the prevalent belief that explainability is an important factor in improving the interaction between AI systems and humans^6,24,39^, the present study showed little evidence for that: while receiving explanations for the model’s predictions might have a small effect on users’ quality perception of the AI-generated advice (Experiment 2b) and their confidence (Experiment 2c), these effects were not consistent, and did not result in higher task performance. Previous work has shown that heatmaps might be too abstract or complex^40^, which may hinder performance. Processing the additional information may have taken as much or even more cognitive resources than receiving no explanations^41^. Additionally, a recent study demonstrated that providing explanations alongside AI advice increased task complexity^42^, which may worsen the cognitive load and negate the anticipated benefits of explanatory aids. Cognitive load refers to the limited mental resources which individuals have for processing information. Under high cognitive load, individuals tend to process information superficially and prioritize easily accessible data^43^. Hence, it is possible that the combination of time pressure and the complex explanations prevented the expected positive effect of reducing overreliance. To give participants the possibility to engage more with explanations, and therefore facilitate their understanding, we removed the time limit. Even though explainable advice in form of salient heatmaps increased people’s tendency for relying on correct advice when participants did not experience time pressure (see Figure S2), it still did not significantly reduce participants’ reliance on incorrect advice. These findings are in line with other research, indicating that people successfully incorporate given explanations into decision-making when they are able to reduce cognitive costs (e.g., when explanations are simple and easy to understand, requiring fewer cognitive resources to process)^44^.

Surprisingly, using a simpler explanation technique to see if complexity contributed to explainability’s limited effectiveness in reducing overreliance did not improve performance but had a more nuanced effect on the advice’s quality rating and participants’ confidence. In Experiment 2c, explainable advice positively affected the quality rating for correct advice only, whereas confidence was higher only for incorrect advice. The first results indicate that receiving a simpler explanation could make it easier for people to detect incorrect advice. In turn, detecting incorrect advice more easily might boost participants’ confidence in their decision to override the incorrect advice. These results are contrary to studies suggesting that explanations also increase the reliance on incorrect AI advice^45^ and further improve trust in AI advice even with low accuracy rates^29^. In our experiment, explainable AI advice did not increase overreliance, however, it also did not result in a better performance either. Overall, explainable AI advice in form of salient heatmaps compared to non-explainable AI advice presented without time pressure only increased people’s reliance on correct advice.

Tailoring explanations to a specific user may be a first step in reducing the perceived complexity of the given explanations^42^ and therefore improving the interaction with AI systems. Customizing explanations to a specific user might go hand in hand with the further implication of using explanations that are easier to understand, which in turn have the potential to decrease cognitive costs^44^. However, more research is needed to fully understand how, and specifically which kind of explainable AI advice affects people’s decision-making.

Overall, the results of the present study support our research question on the source of advice and hypothesis on the accuracy of advice, while failing to fully corroborate our hypothesis on explainable advice. These findings indicate that regardless of the type of advice presented, people showed a strong tendency to follow it. While reliance on high-performing AI decision support systems might result in overall good hiring decisions, and research has shown that NLP models can perform personnel selection tasks as well as humans^46^, none of these systems will be 100% accurate. Making the AI advice more explainable did not strongly influence performance which raises the important question of how overreliance on incorrect AI advice can be prevented, especially in critical tasks such as personnel selection. According to a new EU regulation, AI systems for personnel selection will be considered “high-risk” and will have to undergo strict regulations^47^. As the EU framework will not be applicable until 2024 at the earliest and standards are still under development, exploring solutions to mitigate overreliance on faulty AI advice is essential to implement high-quality and safe AI decision support tools in HRM and other high-stake areas.

### Practical implications

Practical implications stemming from the present study are multifaceted. First, given the consistent findings of reliance on even incorrect advice in this study and numerous others (for instance, see^10,17^), the advice quality becomes a critical factor that should be taken into account in the establishment of robust regulations and standards. Regulatory frameworks, such as those currently being developed by the EU^47^, need to consider the impact of advice quality in users’ interaction with these systems to ensure a safe implementation. For instance, one approach could be displaying AI advice only when it surpasses a certain threshold of system certainty and thereby potentially reducing the risk of incorrect advice. Second, from the users’ perspective, mitigating the risk of relying on any given advice should include careful consideration of how explanations are presented and the level of engagement expected from users. Given the limited effects of explanation methods observed in our study, prompting users to engage analytically with explanations could aid in averting blind acceptance of advice^48^.

### Limitations and future research

The present study had some limitations that provide opportunities for future research. First, participants were aware that making an inaccurate decision did not entail negative real-life consequences. Accordingly, the motivation to perform well may have been limited. Future research could address this issue by including a short justification for the decisions to ensure a more conscientious task performance. Second, the short reviewing time was chosen to ensure external validity, but in practice, recruiters are usually more autonomous when reviewing resumes. Even though infinite reviewing time did not significantly affect the results, further research should investigate the effects of time pressure. Third, in practice, recruiters usually have more discretion when considering selection criteria because suitability is usually based on the applicant’s overall picture. However, we had to enforce strict rules to obtain a clean and comparable performance measure in the experiment. Last, some effects were no longer significant after controlling for covariates, suggesting that these effects may not be stable and differ depending on study characteristics, for instance methodology. Future research should explore these effects more in detail, as understanding the robustness of effects is crucial.

## Conclusions

In conclusion, the findings of this study are important for both research and practice, as they show that the algorithms’ accuracy is the key factor for the successful deployment of AI-enabled decision support systems in personnel selection. On a more global level, the fact that making the systems’ predictions more explainable did not reduce participants’ overreliance on incorrect advice emphasizes the complexity of human-AI interaction and the need for regulations and high-quality standards. Future research should focus on how AI advice should be presented to prevent users from blindly following it.

### Supplementary Information

Supplementary Information.

## Data Availability

The study’s preregistration, data, survey material, and R-Script will be made available online upon publication: https://osf.io/dfwy6/.
