# Supplementary material for: Explainability does not mitigate the negative impact of incorrect AI advice in a personnel selection task
Source: Sci Rep. 2024 Apr 28;14:9736. doi: 10.1038/s41598-024-60220-5 (PMC11056364; doi:10.1038/s41598-024-60220-5)
Supplement: Supplementary file 1 — Supplementary Information. [file 41598_2024_60220_MOESM1_ESM.docx]

**Supplemental Material**

Explainability does not mitigate the negative impact of incorrect AI advice in a personnel selection task

**Material conception**

For our study, 18 different resume layouts were created. Previously published resumes from LinkedIn and Xing were used as templates for adequate professional experience and academic achievements. The content was adapted so that 50% of the applicants met the selection criteria and would therefore be suitable for the position (Head of Quality Management). To avoid potential gender bias, each resume presented the first name using only the initial letter and did not use any terms that could be used to draw conclusions about the applicant's gender. Dates of birth were randomized via Microsoft Excel. In addition, 18 telephone numbers as well as e-mail addresses were created and randomly assigned to the resumes via Microsoft Excel. Address assignment was also randomized. Since the position was Head of Quality Management of an automotive company in Munich (Germany), the addresses of the applicants were based thereon. Most of the resumes, including those in the exercise block, were each assigned an address from Munich, the surrounding area, and automotive locations. The addresses were generated systematically. In each case, the geographical center of the location was determined using Google Maps, the geographically closest address was selected and randomly assigned to a resume. The work locations were manually adjusted to the place of residence afterwards. The employer's existing locations were considered to maintain external validity.

**Selection criteria**

*Original German version*

- abgeschlossenes Studium im technischen Bereich z.B. Produktion & Management, Maschinenbau, Wirtschaftsingenieurwesen, Fahrzeugtechnik, Verfahrenstechnik o.Ä.
- Mind. 8 Jahre praxisbezogene Berufserfahrung in dem Bereich Qualitätsmanagement
- Mind. 3 Jahre Erfahrung in der Personalführung (Leitung, Bereichsleitung, Teamleitung oder Projektleitung mit Führungserfahrung)
- Erfahrung im QM der Automobilindustrie (bei einem Hersteller [OEM] und/oder bei einem Zulieferer)
- Zertifizierung zum internen Auditor und VDA 6.3 Prozessauditor (nur Personen mit diesen Zertifizierungen dürfen interne Audits und VDA 6.3 Prozessaudits durchführen)
- verhandlungssichere Deutschkenntnisse | sehr gute Englischkenntnisse
- sehr gute MS Office und SAP Kenntnisse

*Translated English version*

- Degree in technical field e.g., production & management, mechanical engineering, industrial engineering, automotive engineering, process engineering or similar.
- At least 8 years of practical work experience in the field of quality management
- At least 3 years of experience in personnel management (management, division management, team management or project management with leadership experience)
- Experience in QM in the automotive industry (at a manufacturer [OEM] and/or at a supplier)
- Certification as internal auditor and VDA 6.3 process auditor (only persons with these certifications are allowed to perform internal audits and VDA 6.3 process audits)
- German language skills: business fluent | English language skills: very good
- very good MS Office and SAP knowledge

**Detailed procedure**

All criteria had to be met by each candidate to be considered suitable for the position. To ensure that participants understood and remembered the selection criteria, they were quizzed on them before the experiment began. Participation was terminated if the selection criteria were incorrectly identified after two attempts.

After reviewing the resumes, participants also completed a survey assessing demographics, experience in HRM, and attitude towards AI. Based on theoretical relevance, experience in HRM was included as a control variable (for a review on performance, see [1]). Attitude towards AI was included as a control variable as it emerged as an important factor in previous research [2]. Additional potentially relevant control variables were measured depending on the sample: In Experiments 1a, 2a, 2b, and 2c needs for competence and autonomy (German short version of the Basic Psychological Need Satisfaction and Frustration Scale (BPNSNF), [3]) and self-reported affinity for technology (German short version of the Affinity for Technology Interaction Scale (ATI), [4]). The needs for competence and autonomy were included based on various previous findings highlighting the predictive role of both needs on performance (for a meta-analysis, see [5]). Affinity for technology interaction significantly influenced people’s AI-related behavior [6, 7]. Participants in Study 2a, 2b, and 2c were asked to rate their AI knowledge (Gaube et al., 2021). AI knowledge was included as it emerged as a relevant factor in a similar study [8]. In Study 1b, beliefs in professional autonomy [9] and professional identification [10] replaced the BPNSNF scales [3] used in Experiment 1a to adjust for experience.

**Variables**

Across all experiments, several additional variables used as control variables in the regression models were measured.

A) *Need for competence*: Four items (e.g., “I feel competent to achieve my goals.”) were answered on a Likert scale from 1 (*completely disagree*) to 5 (*completely agree*); Cronbach's α ≥ 0.73 (0.73 – 0.76)

B) *Need for autonomy*: Four items (e.g., “I feel that my decisions reflect what I really want.”) were answered on a Likert scale from 1 (*completely disagree*) to 5 (*completely agree*); Cronbach's α ≥ 0.62 (0.62 – 0.72)

C) *Belief in professional autonomy*: Four items (e.g., "Individual HRM employees

should make their own decisions regarding what is to be done in their work." were answered on a Likert scale from 1 (*strongly disagree*) to 7 (*strongly agree*); Cronbach's α = 0.67.

D) *Professional identification*: Five items (e.g., "In general, when someone praises HRM employees, it feels like a personal compliment.") were answered on a Likert scale from 1 (*strongly disagree*) to 7 (*strongly agree*); Cronbach's α = 0.72.

E) *Affinity for technology*: Four items (e.g., “It is enough for me that a technical system works; I don’t care how or why”) were answered on a Likert scale from 1 (*completely disagree*) to 6 (*completely agree*); Cronbach's α ≥ 0.85 (0.85 – 0.88).

F) *Attitude toward AI*: Six items (e.g., "How much do you agree with the following statements? AI will make most people's lives better; AI is dangerous to society.) were answered on a Likert scale from 1 (*strongly disagree*) to 7 (*strongly agree*); Cronbach's α = 0.73 (0.73 – 0.84).

G) *Self-reported AI knowledge*: One item ("How would you consider your own general knowledge of artificial intelligence (AI)?” was answered on a scale from 1 (*I have no knowledge*) to 5 (*Expert knowledge*).


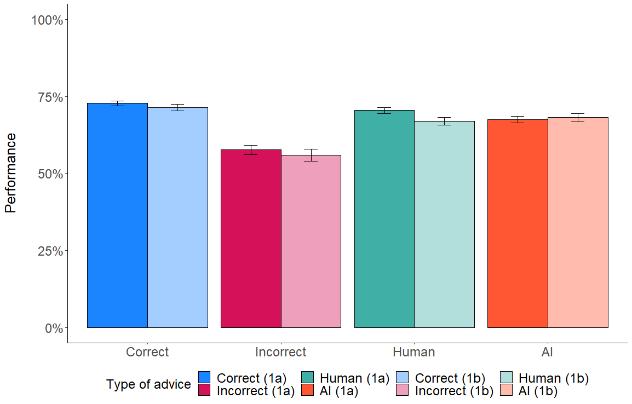


**Figure S1.** *Performance rates by type of advice of Experiment 1a (students) and 1b (HRM employees).* The error bars represent standard errors**.**

**
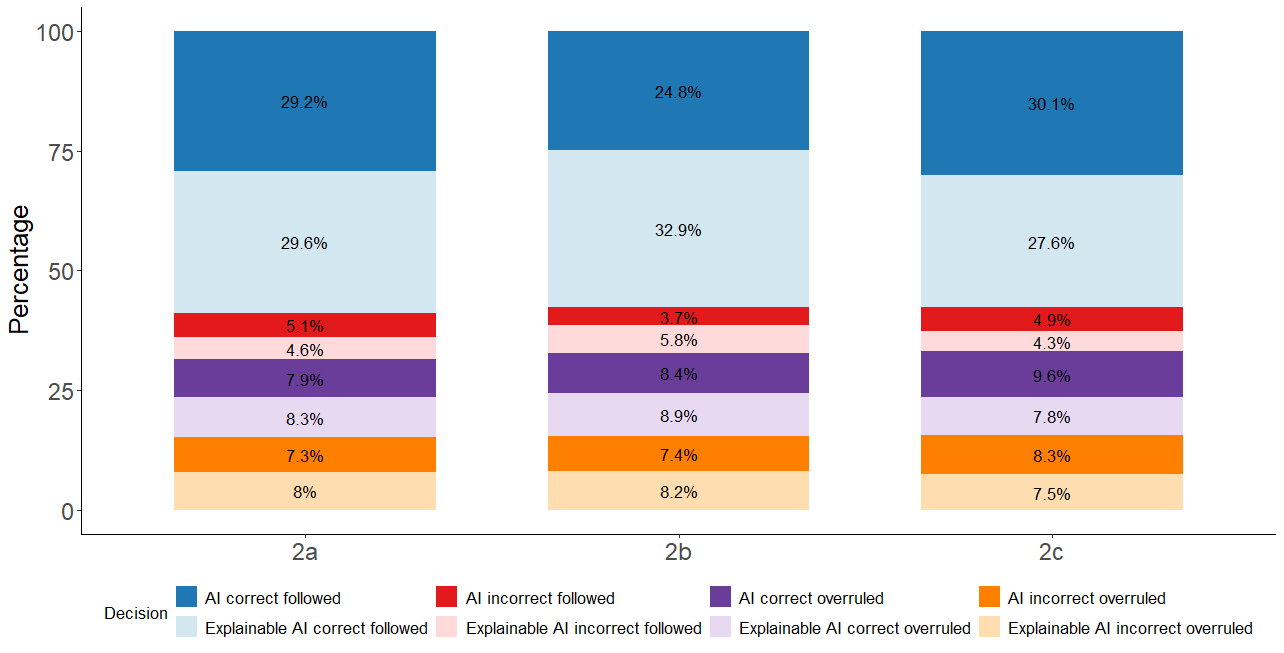
**

**Figure S2**. *Percentage overview of the different combinations of type of advice and participant decisions for Experiments 2a – 2c*. Correct followed = Presentation of correct advice and participants’ decision was correct, Incorrect followed = Presentation of incorrect advice and participants’ decision was incorrect, Correct overruled = Presentation of correct advice but participants’ decision was incorrect, Incorrect overruled = Presentation of incorrect advice but participants’ decision was correct.

**Experiment 1a: Mixed multilevel regression models with covariates**

**Table S1**

*Logistic mixed multilevel regressions for performance*

| *Predictors* | *Odds Ratio* | *SE* | *95% CI* | *z* | *p* |
| --- | --- | --- | --- | --- | --- |
| Intercept | 0.33 | 0.20 | 0.10 – 1.08 | -1.84 | .066 |
| Accuracy [Correct] | 2.81 | 0.44 | 2.07 – 3.83 | 6.58 | **<.001** |
| Source [Human] | 1.44 | 0.29 | 0.98 – 2.13 | 1.86 | .063 |
| Professional experience (years) | 1.01 | 0.03 | 0.95 – 1.08 | 0.29 | .770 |
| Attitude toward AI | 1.12 | 0.09 | 0.95 – 1.32 | 1.38 | .169 |
| Need for autonomy | 1.04 | 0.09 | 0.87 – 1.24 | 0.42 | .677 |
| Need for competence | 1.06 | 0.13 | 0.84 – 1.34 | 0.51 | .608 |
| AI-Affinity | 0.97 | 0.05 | 0.88 – 1.07 | -0.57 | .570 |
| AI-Knowledge | 1.11 | 0.07 | 0.99 – 1.25 | 1.71 | .087 |
| Source x Accuracy | 0.75 | 0.17 | 0.48 – 1.16 | -1.30 | .195 |
| *Notes*. *SE* = standard error; *p* = probability of committing a Type I error; random effects: σ^2^ =3.29, τ_00 ID_ = 0.08, τ_00 CV_= 0.34, ICC = 0.11, N_ID_ = 125, N_CV_= 16, Observations = 2000, Marginal R^2^ = 0.05 / Conditional R^2^ = 0.16. OR>1 variable associated with higher odds for correct decision; OR<1 variable associated with lower odds for correct decision, OR=1 variable does not affect outcome odds. | | | | | |

**Table S2**

*Linear mixed multilevel regressions for advice quality rating*

| *Predictors* | *β* | *SE* | *95% CI* | *t* | *p* |
| --- | --- | --- | --- | --- | --- |
| Intercept | 2.73 | 1.03 | 0.71 – 4.75 | 2.66 | **.008** |
| Accuracy [Correct] | 0.39 | 0.08 | 0.23 – 0.54 | 4.92 | **<.001** |
| Source [Human] | 0.21 | 0.22 | -0.23 – 0.65 | 0.92 | .359 |
| Professional experience (years) | 0.07 | 0.06 | -0.05 – 0.18 | 1.15 | .251 |
| Attitude toward AI | 0.01 | 0.15 | -0.28 – 0.31 | 0.09 | .926 |
| Need for autonomy | 0.11 | 0.16 | -0.21 – 0.43 | 0.69 | .492 |
| Professional identification | 0.07 | 0.21 | -0.35 – 0.49 | 0.32 | .753 |
| AI-Affinity | 0.02 | 0.09 | -0.16 – 0.19 | 0.18 | .855 |
| AI-Knowledge | -0.03 | 0.11 | -0.24 – 0.18 | -0.28 | .776 |
| Source x Accuracy | 0.01 | 0.11 | -0.21 – 0.23 | 0.11 | .914 |
| *Notes*. *SE* = standard error; *p* = probability of committing a Type I error; random effects: σ^2^ =1.16, τ_00 ID_ = 1.21, τ_00 CV_= 0.01, ICC = 0.51, N_ID_ = 125, N_CV_= 16, Observations = 2000, Marginal R^2^ = 0.03 / Conditional R^2^ = 0.53. The regression estimate *β* indicates how much the mean quality rating changes given a one-unit shift in the predictor while holding other predictors in the model constant | | | | | |

**Table S3**

*Linear mixed multilevel regressions for confidence in one’s decision*

| *Predictors* | *β* | *SE* | *95% CI* | *t* | *p* |
| --- | --- | --- | --- | --- | --- |
| Intercept | 3.44 | 0.64 | 2.19 – 4.69 | 5.39 | **<.001** |
| Accuracy [Correct] | 0.13 | 0.07 | -0.02 – 0.27 | 1.73 | .084 |
| Source [Human] | 0.41 | 0.15 | 0.11 – 0.70 | 2.69 | **.007** |
| Professional experience (years) | 0.05 | 0.04 | -0.02 – 0.12 | 1.39 | .165 |
| Attitude toward AI | 0.10 | 0.09 | -0.08 – 0.28 | 1.09 | .277 |
| Need for autonomy | 0.33 | 0.10 | 0.13 – 0.52 | 3.22 | **.001** |
| Need for competence | -0.12 | 0.13 | -0.38 – 0.14 | -0.93 | .354 |
| AI-Affinity | 0.06 | 0.06 | -0.05 – 0.17 | 1.11 | .269 |
| AI-Knowledge | 0.06 | 0.07 | -0.07 – 0.19 | 0.89 | .373 |
| Source x Accuracy | -0.03 | 0.11 | -0.23 – 0.18 | -0.26 | .795 |
| *Notes*. *SE* = standard error; *p* = probability of committing a Type I error; random effects: σ^2^ =1.03, τ_00 ID_ = 0.42, τ_00 CV_= 0.04, ICC = 0.31, N_ID_ = 125, N_CV_= 16, Observations = 2000, Marginal R^2^ = 0.06 / Conditional R^2^ = 0.35. The regression estimate *β* indicates how much the mean quality rating changes given a one-unit shift in the predictor while holding other predictors in the model constant. | | | | | |

**Experiment 1b: Mixed multilevel regression models with covariates**

**Table S4**

*Logistic mixed multilevel regressions for performance*

| *Predictors* | *Odds Ratio* | *SE* | *95% CI* | *z* | *p* |
| --- | --- | --- | --- | --- | --- |
| Intercept | 1.34 | 0.53 | 0.62 – 2.90 | 0.75 | .454 |
| Accuracy [Correct] | 2.42 | 0.34 | 1.83 – 3.19 | 6.25 | **<.001** |
| Source [Human] | 1.15 | 0.19 | 0.83 – 1.59 | 0.82 | .413 |
| Professional experience (years) | 0.98 | 0.01 | 0.97 – 1.00 | -1.58 | .114 |
| Attitude toward AI | 1.03 | 0.06 | 0.92 – 1.15 | 0.52 | .602 |
| Need for autonomy | 1.00 | 0.05 | 0.92 – 1.10 | 0.09 | .931 |
| Professional identification | 1.00 | 0.04 | 0.92 – 1.09 | 0.07 | .947 |
| AI-Knowledge | 0.94 | 0.04 | 0.86 – 1.02 | -1.56 | .118 |
| Source x Accuracy | 0.76 | 0.15 | 0.52 – 1.12 | -1.38 | .167 |
| *Notes*. *SE* = standard error; *p* = probability of committing a Type I error; random effects: σ^2^ =3.29, τ_00 ID_ = 0.00, τ_00 CV_= 0.43, ICC = 0.11, N_ID_ = 162, N_CV_= 16, Observations = 2592, Marginal R^2^ = 0.03 / Conditional R^2^ = 0.14. OR>1 variable associated with higher odds for correct decision; OR<1 variable associated with lower odds for correct decision, OR=1 variable does not affect outcome odds. | | | | | |

**Table S5**

*Linear mixed multilevel regressions for advice quality rating*

| *Predictors* | *β* | *SE* | *95% CI* | *t* | *p* |
| --- | --- | --- | --- | --- | --- |
| Intercept | 3.64 | 0.65 | 2.37 – 4.92 | 5.61 | **<.001** |
| Accuracy [Correct] | 0.41 | 0.07 | 0.27 – 0.54 | 5.91 | **<.001** |
| Source [Human] | -0.25 | 0.18 | -0.60 – 0.11 | -1.38 | .169 |
| Professional experience (years) | -0.01 | 0.02 | -0.05 – 0.03 | -0.44 | .658 |
| Attitude toward AI | -0.14 | 0.10 | -0.34 – 0.07 | -1.34 | .182 |
| Need for autonomy | 0.14 | 0.09 | -0.03 – 0.31 | 1.65 | .099 |
| Professional identification | 0.09 | 0.08 | -0.07 – 0.25 | 1.14 | .255 |
| AI-Knowledge | 0.01 | 0.08 | -0.14 – 0.17 | 0.19 | .853 |
| Source x Accuracy | 0.02 | 0.10 | -0.17 – 0.20 | 0.18 | .859 |
| *Notes*. *SE* = standard error; *p* = probability of committing a Type I error; random effects: σ^2^ =1.10, τ_00 ID_ = 1.05, τ_00 CV_= 0.00, ICC = 0.49, N_ID_ = 162, N_CV_= 16, Observations = 2592, Marginal R^2^ = 0.05 / Conditional R^2^ = 0.51. The regression estimate *β* indicates how much the mean quality rating changes given a one-unit shift in the predictor while holding other predictors in the model constant. | | | | | |

**Table S6**

*Linear mixed multilevel regressions for confidence in one’s decision*

| *Predictors* | *β* | *SE* | *95% CI* | *t* | *p* |
| --- | --- | --- | --- | --- | --- |
| Intercept | 5.25 | 0.41 | 4.46 – 6.05 | 12.96 | **<.001** |
| Accuracy [Correct] | 0.18 | 0.07 | 0.05 – 0.31 | 2.63 | **.008** |
| Source [Human] | -0.05 | 0.12 | -0.30 – 0.19 | -0.42 | .672 |
| Professional experience (years) | -0.03 | 0.01 | -0.05 – -0.01 | -2.58 | **.010** |
| Attitude toward AI | 0.05 | 0.06 | -0.08 – 0.18 | 0.77 | .439 |
| Need for autonomy | -0.09 | 0.05 | -0.19 – 0.02 | -1.65 | .099 |
| Professional identification | 0.02 | 0.05 | -0.07 – 0.12 | 0.50 | .620 |
| AI-Knowledge | 0.03 | 0.05 | -0.07 – 0.12 | 0.56 | .574 |
| Source x Accuracy | 0.09 | 0.09 | -0.10 – 0.27 | 0.93 | .353 |
| *Notes*. *SE* = standard error; *p* = probability of committing a Type I error; random effects: σ^2^ =1.04, τ_00 ID_ = 0.36, τ_00 CV_= 0.02, ICC = 0.27, N_ID_ = 162, N_CV_= 16, Observations = 2592, Marginal R^2^ = 0.03 / Conditional R^2^ = 0.29. The regression estimate *β* indicates how much the mean quality rating changes given a one-unit shift in the predictor while holding other predictors in the model constant | | | | | |

**Experiment 2a: Mixed multilevel regression models with covariates**

**Table S7**

*Logistic mixed multilevel regressions for performance*

| *Predictors* | *Odds Ratio* | *SE* | *95% CI* | *z* | *p* |
| --- | --- | --- | --- | --- | --- |
| Intercept | 1.42 | 0.54 | 0.68 – 2.98 | 0.93 | .353 |
| Accuracy [Correct] | 2.85 | 0.36 | 2.23 – 3.64 | 8.33 | **<.001** |
| Explainability [Explainable] | 1.26 | 0.19 | 0.94 – 1.70 | 1.56 | .119 |
| Professional experience (years) | 0.97 | 0.03 | 0.92 – 1.03 | -0.96 | .336 |
| Attitude toward AI | 1.06 | 0.05 | 0.97 – 1.15 | 1.27 | .204 |
| Need for autonomy | 0.95 | 0.06 | 0.84 – 1.08 | -0.76 | .448 |
| Need for competence | 1.01 | 0.07 | 0.88 – 1.17 | 0.17 | .865 |
| Affinity for Technology | 1.00 | 0.04 | 0.93 – 1.07 | -0.07 | .945 |
| AI-Knowledge | 0.98 | 0.05 | 0.90 – 1.08 | -0.36 | .717 |
| Explainability x Accuracy | 0.76 | 0.13 | 0.54 – 1.07 | -1.56 | .119 |
| *Notes*. *SE* = standard error; *p* = probability of committing a Type I error; random effects: σ^2^ =3.29, τ_00 ID_ = 0.04, τ_00 CV_= 0.39, ICC = 0.12, N_ID_ = 214, N_CV_= 16, Observations = 3424, Marginal R^2^ = 0.04 / Conditional R^2^ = 0.15. OR>1 variable associated with higher odds for correct decision; OR<1 variable associated with lower odds for correct decision, OR=1 variable does not affect outcome odds. | | | | | |

**Table S8**

*Linear mixed multilevel regressions for advice quality rating*

| *Predictors* | *β* | *SE* | *95% CI* | *t* | *p* |
| --- | --- | --- | --- | --- | --- |
| Intercept | 2.32 | 0.57 | 1.21 – 3.44 | 4.08 | **<.001** |
| Accuracy [Correct] | 0.59 | 0.06 | 0.48 – 0.71 | 9.98 | **<.001** |
| Explainability [Explainable] | 0.20 | 0.16 | -0.12 – 0.52 | 1.21 | .227 |
| Professional experience (years) | -0.09 | 0.05 | -0.19 – 0.00 | -1.90 | .058 |
| Attitude toward AI | 0.18 | 0.07 | 0.04 – 0.32 | 2.44 | **.015** |
| Need for autonomy | -0.09 | 0.11 | -0.31 – 0.12 | -0.84 | .400 |
| Need for competence | 0.10 | 0.12 | -0.14 – 0.34 | 0.84 | .402 |
| Affinity for Technology | 0.13 | 0.06 | 0.01 – 0.25 | 2.10 | **.036** |
| AI-Knowledge | -0.00 | 0.08 | -0.16 – 0.16 | -0.06 | .955 |
| Explainability x Accuracy | 0.01 | 0.08 | -0.15 – 0.17 | 0.12 | .903 |
| *Notes*. *SE* = standard error; *p* = probability of committing a Type I error; random effects: σ^2^ =1.12, τ_00 ID_ = 1.08, τ_00 CV_= 0.01, ICC = 0.49, N_ID_ = 214, N_CV_= 16, Observations = 3424, Marginal R^2^ = 0.07 / Conditional R^2^ = 0.53. The regression estimate *β* indicates how much the mean quality rating changes given a one-unit shift in the predictor while holding other predictors in the model constant. | | | | | |

**Table S9**

*Linear mixed multilevel regressions for confidence in one’s decision*

| *Predictors* | *β* | *SE* | *95% CI* | *t* | *p* |
| --- | --- | --- | --- | --- | --- |
| Intercept | 3.05 | 0.43 | 2.20 – 3.89 | 7.08 | **<.001** |
| Accuracy [Correct] | 0.24 | 0.06 | 0.11 – 0.36 | 3.71 | **<.001** |
| Explainability [Explainable] | -0.00 | 0.13 | -0.26 – 0.26 | -0.01 | .988 |
| Professional experience (years) | 0.07 | 0.04 | 0.00 – 0.14 | 2.04 | **.041** |
| Attitude toward AI | 0.07 | 0.05 | -0.04 – 0.17 | 1.21 | .225 |
| Need for autonomy | 0.02 | 0.08 | -0.14 – 0.19 | 0.26 | .797 |
| Need for competence | 0.36 | 0.09 | 0.18 – 0.54 | 3.95 | **<.001** |
| AI-Affinity | 0.09 | 0.05 | -0.00 – 0.18 | 1.87 | .061 |
| AI-Knowledge | -0.07 | 0.06 | -0.19 – 0.05 | -1.09 | .276 |
| Explainability x Accuracy | 0.13 | 0.09 | -0.05 – 0.30 | 1.40 | .161 |
| *Notes*. *SE* = standard error; *p* = probability of committing a Type I error; random effects: σ^2^ =1.28, τ_00 ID_ = 0.57, τ_00 CV_= 0.04, ICC = 0.32, N_ID_ = 214, N_CV_= 16, Observations = 3424, Marginal R^2^ = 0.07 / Conditional R^2^ = 0.37. The regression estimate *β* indicates how much the mean quality rating changes given a one-unit shift in the predictor while holding other predictors in the model constant. | | | | | |

**Experiment 2b: Mixed multilevel regression models with covariates**

**Table S10**

*Logistic mixed multilevel regressions model for performance*

| *Predictors* | *Odds Ratio* | *SE* | *95% CI* | *z* | *p* |
| --- | --- | --- | --- | --- | --- |
| Intercept | 2.48 | 1.86 | 0.78 – 10.27 | 1.59 | **.112** |
| Accuracy [Correct] | 1.68 | 0.34 | 1.13 – 2.50 | 2.59 | **.010** |
| Explainability [Explainable] | 0.75 | 0.17 | 0.48 – 1.18 | -1.23 | .218 |
| Professional experience (years) | 0.99 | 0.04 | 0.93 – 1.08 | -0.16 | .869 |
| Attitude toward AI | 1.01 | 0.08 | 0.87 – 1.17 | -0.17 | .865 |
| Need for autonomy | 1.04 | 0.13 | 0.82 – 1.33 | 0.33 | .738 |
| Need for competence | 0.81 | 0.11 | 0.62 – 1.07 | -1.49 | .137 |
| Affinity for Technology | 1.03 | 0.06 | 0.91 – 1.16 | 0.44 | .659 |
| AI-Knowledge | 1.08 | 0.08 | 0.94 – 1.24 | 1.13 | .257 |
| Explainability x Accuracy | 1.62 | 0.43 | 0.96 – 2.73 | 1.80 | .072 |
| *Notes*. *SE* = standard error; *p* = probability of committing a Type I error; random effects: σ^2^ =3.29, τ_00 ID_ = 0.07, τ_00 CV_= 0.35, ICC = 0.11, N_ID_ = 95, N_CV_= 16, Observations = 1520, Marginal R^2^ = 0.04 / Conditional R^2^ = 0.15. OR>1 variable associated with higher odds for correct decision; OR<1 variable associated with lower odds for correct decision, OR=1 variable does not affect outcome odds. | | | | | |

**Table S11**

*Linear mixed multilevel regressions model for advice quality rating*

| *Predictors* | *β* | *SE* | *95% CI* | *t* | *p* |
| --- | --- | --- | --- | --- | --- |
| Intercept | 0.50 | 1.02 | -1.50 – 2.50 | 0.49 | .627 |
| Accuracy [Correct] | 0.81 | 0.10 | 0.62 – 1.00 | 8.30 | **<.001** |
| Explainability [Explainable] | 0.43 | 0.24 | -0.05 – 0.91 | 1.77 | .077 |
| Professional experience (years) | -0.01 | 0.07 | -0.15 – 0.13 | -0.11 | .911 |
| Attitude toward AI | 0.44 | 0.13 | 0.20 – 0.69 | 3.50 | **<.001** |
| Need for autonomy | -0.18 | 0.20 | -0.58 – 0.22 | -0.89 | .374 |
| Need for competence | 0.36 | 0.23 | -0.08 – 0.81 | 1.60 | .109 |
| Affinity for Technology | 0.07 | 0.10 | -0.13 – 0.27 | 0.67 | .505 |
| AI-Knowledge | 0.01 | 0.11 | -0.22 – 0.23 | 0.05 | .958 |
| Explainability x Accuracy | -0.25 | 0.13 | -0.51 – 0.00 | -1.94 | .052 |
| *Notes*. *SE* = standard error; *p* = probability of committing a Type I error; random effects: σ^2^ =1.20, τ_00 ID_ = 1.06, τ_00 CV_= 0.00, ICC = 0.47, N_ID_ = 95, N_CV_= 16, Observations = 1520, Marginal R^2^ = 0.12 / Conditional R^2^ = 0.53. The regression estimate *β* indicates how much the mean quality rating changes given a one-unit shift in the predictor while holding other predictors in the model constant. | | | | | |

**Table S12**

*Linear mixed multilevel regressions model for confidence in one’s decision*

| *Predictors* | *β* | *SE* | *95% CI* | *t* | *p* |
| --- | --- | --- | --- | --- | --- |
| Intercept | 5.01 | 0.71 | 3.61 – 6.41 | 7.04 | **<.001** |
| Accuracy [Correct] | 0.16 | 0.08 | 0.00 – 0.33 | 2.00 | **.045** |
| Explainability [Explainable] | 0.18 | 0.18 | -0.17 – 0.52 | 1.01 | .313 |
| Professional experience (years) | 0.01 | 0.05 | -0.08 – 0.11 | 0.24 | .807 |
| Attitude toward AI | 0.06 | 0.09 | -0.11 – 0.23 | 0.67 | .503 |
| Need for autonomy | -0.14 | 0.14 | -0.42 – 0.14 | -0.98 | .327 |
| Need for competence | 0.18 | 0.16 | -0.13 – 0.49 | 1.13 | .257 |
| Affinity for Technology | -0.06 | 0.07 | -0.20 – 0.08 | -0.88 | .381 |
| AI-Knowledge | 0.04 | 0.08 | -0.12 – 0.20 | 0.49 | .623 |
| Explainability x Accuracy | -0.18 | 0.11 | -0.40 – 0.03 | -1.67 | .095 |
| *Notes*. *SE* = standard error; *p* = probability of committing a Type I error; random effects: σ^2^ =0.85, τ_00 ID_ = 0.50, τ_00 CV_= 0.01, ICC = 0.38, N_ID_ = 95, N_CV_= 16, Observations = 1520, Marginal R^2^ = 0.02 / Conditional R^2^ = 0.39. The regression estimate *β* indicates how much the mean quality rating changes given a one-unit shift in the predictor while holding other predictors in the model constant. | | | | | |

**Experiment 2c: Mixed multilevel regression models with covariates**

**Table S13**

*Logistic mixed multilevel regressions model for performance*

| *Predictors* | *Odds Ratios* | *SE* | *95% CI* | *z* | *p* |
| --- | --- | --- | --- | --- | --- |
| Intercept | 2.01 | 0.78 | 0.94 – 4.28 | 1.81 | .070 |
| Accuracy [Correct] | 2.08 | 0.26 | 1.63 – 2.64 | 5.91 | **<.001** |
| Explainability [Explainable] | 1.07 | 0.17 | 0.79 – 1.45 | 0.42 | .671 |
| Professional experience (years) | 0.97 | 0.02 | 0.94 – 1.01 | -1.46 | .143 |
| Attitude toward AI | 1.03 | 0.06 | 0.92 – 1.15 | 0.52 | .603 |
| Need for autonomy | 0.93 | 0.07 | 0.80 – 1.08 | -0.98 | .329 |
| Need for competence | 1.00 | 0.08 | 0.85 – 1.17 | -0.03 | .975 |
| Affinity for Technology | 1.01 | 0.04 | 0.94 – 1.10 | 0.35 | .723 |
| AI-Knowledge | 0.98 | 0.05 | 0.89 – 1.09 | -0.35 | .729 |
| Explainability x Accuracy | 1.05 | 0.19 | 0.73 – 1.49 | 0.25 | .804 |
| *Notes*. *SE* = standard error; *p* = probability of committing a Type I error; random effects: σ^2^ =3.29, τ_00 ID_ = 0.07, τ_00 CV_= 0.43, ICC = 0.13, N_ID_ = 208, N_CV_= 16, Observations = 3328, Marginal R^2^ = 0.03 / Conditional R^2^ = 0.16. OR>1 variable associated with higher odds for correct decision; OR<1 variable associated with lower odds for correct decision, OR=1 variable does not affect outcome odds. | | | | | |

**Table S14**

*Linear mixed multilevel regressions model for advice quality rating*

| *Predictors* | *β* | *SE* | *95% CI* | *t* | *p* |
| --- | --- | --- | --- | --- | --- |
| Intercept | 2.16 | 0.57 | 1.05 – 3.27 | 3.81 | **<.001** |
| Accuracy [Correct] | 0.60 | 0.06 | 0.48 – 0.72 | 9.61 | **<.001** |
| Explainability [Explainable] | 0.07 | 0.17 | -0.26 – 0.39 | 0.40 | .689 |
| Professional experience (years) | -0.01 | 0.03 | -0.07 – 0.05 | -0.38 | .704 |
| Attitude toward AI | 0.39 | 0.10 | 0.21 – 0.58 | 4.11 | **<.001** |
| Need for autonomy | -0.28 | 0.12 | -0.52 – -0.03 | -2.23 | **.026** |
| Need for competence | 0.27 | 0.13 | 0.01 – 0.53 | 2.02 | **.043** |
| Affinity for Technology | -0.02 | 0.07 | -0.14 – 0.11 | -0.24 | .812 |
| AI-Knowledge | -0.13 | 0.09 | -0.30 – 0.04 | -1.54 | .125 |
| Explainability x Accuracy | 0.24 | 0.09 | 0.06 – 0.42 | 2.61 | **.009** |
| *Notes*. *SE* = standard error; *p* = probability of committing a Type I error; random effects: σ^2^ =1.29, τ_00 ID_ = 1.10, τ_00 CV_= 0.01, ICC = 0.46, N_ID_ = 208, N_CV_= 16, Observations = 3328, Marginal R^2^ = 0.10 / Conditional R^2^ = 0.52. The regression estimate *β* indicates how much the mean quality rating changes given a one-unit shift in the predictor while holding other predictors in the model constant. | | | | | |

**Table S15**

*Linear mixed multilevel regressions for confidence in one’s decision*

| *Predictors* | *β* | *SE* | *95% CI* | *t* | *p* |
| --- | --- | --- | --- | --- | --- |
| Intercept | 3.51 | 0.40 | 2.74 – 4.29 | 8.88 | **<.001** |
| Accuracy [Correct] | 0.43 | 0.06 | 0.32 – 0.54 | 7.57 | **<.001** |
| Explainability [Explainable] | 0.38 | 0.12 | 0.15 – 0.62 | 3.15 | **.002** |
| Professional experience (years) | 0.06 | 0.02 | 0.02 – 0.10 | 2.83 | **.005** |
| Attitude toward AI | 0.21 | 0.07 | 0.08 – 0.34 | 3.17 | **.002** |
| Need for autonomy | 0.13 | 0.09 | -0.04 – 0.30 | 1.51 | .131 |
| Need for competence | -0.07 | 0.09 | -0.25 – 0.12 | -0.71 | .477 |
| AI-Affinity | 0.11 | 0.05 | 0.02 – 0.20 | 2.47 | **.014** |
| AI-Knowledge | -0.02 | 0.06 | -0.13 – 0.10 | -0.28 | .780 |
| Explainability x Accuracy | -0.38 | 0.08 | -0.54 – -0.21 | -4.53 | **<.001** |
| *Notes*. *SE* = standard error; *p* = probability of committing a Type I error; random effects: σ^2^ =1.07, τ_00 ID_ = 0.494, τ_00 CV_= 0.05, ICC = 0.34, N_ID_ = 208, N_CV_= 16, Observations = 3328, Marginal R^2^ = 0.07 / Conditional R^2^ = 0.38. The regression estimate *β* indicates how much the mean quality rating changes given a one-unit shift in the predictor while holding other predictors in the model constant. | | | | | |

| **Table S16** |  |  |  |  |  |
| --- | --- | --- | --- | --- | --- |
| *Mean and standard deviations for each combination of advice and participant decision across all experiments* | | | | | |
|  | *Correct followed* | *Incorrect followed* | *Correct overruled* | *Incorrect overruled* | *Without advice* |
| **Quality** |  |  |  |  |  |
| #1a | 4.33 (1.38) | 4.33 (1.37) | 2.96 (1.19) | 2.99 (1.18) | -- |
| #1b | 4.57 (1.39) | 4.54 (1.29) | 3.22 (1.24) | 3.15 (1.29) | -- |
| #2a | 4.55 (1.43) | 4.48 (1.46) | 3.21 (1.34) | 3.14 (1.25) | -- |
| #2b | 4.83 (1.40) | 4.90 (1.46) | 3.29 (1.34) | 3.13 (1.31) | -- |
| #2c | 4.62 (1.47) | 4.57 (1.50) | 3.13 (1.33) | 2.97 (1.39) | -- |
|  |  |  |  |  |  |
| **Confidence** |  |  |  |  |  |
| #1a | 5.45 (1.22) | 5.10 (1.30) | 4.82 (1.26) | 5.18 (1.25) | 5.05 (1.47) |
| #1b | 5.60 (1.17) | 5.26 (1.27) | 5.07 (1.16) | 5.20 (1.18) | 5.27 (1.36) |
| #2a | 5.52 (1.33) | 5.02 (1.53) | 4.75 (1.46) | 5.06 (1.41) | 5.17 (1.38) |
| #2b | 5.75 (1.09) | 5.56 (1.37) | 5.16 (1.25) | 5.56 (1.01) | 5.38 (1.24) |
| #2c | 5.53 (1.23) | 5.19 (1.34) | 4.90 (1.34) | 5.10 (1.34) | 5.18 (1.43) |

**Deviation from the Preregistration**

The in Experiment 2a-2c preregistered advice quality evaluation [11] (RQ6: Does explainability (vs. non-explainability) of advice affect a more comprehensive advice quality evaluation at the end of the experiment?) was not included in the final paper as we decided to prioritize the most important or relevant hypothesis and research questions for the study.

**References**

1. Quińones, M. A., Ford, J. K. & Teachout, M. S. The Relationship Between Work Experience and Job Performance: A Conceptual and Meta-Analytic Review. *Pers. Psychol.* **48**, 887–910 (1995).

2. Gaube, S. *et al.* Non-task expert physicians benefit from correct explainable AI advice when reviewing X-rays. *Sci. Rep.* **13**, https://doi.org/10.1038/s41598-023-28633-w (2023).

3. Heissel, A., Pietrek, A., Flunger, B., Fydrich, T., Rapp, M. A., Heinzel, S., & Vansteenkiste, M. The validation of the German Basic Psychological Need Satisfaction and Frustration Scale in the Context of Mental Health. *Eur. J. Health Psychol.* **25**, 119–132. <https://doi.org/10.1027/2512-8442/a000017> (2018).

4. Franke, T., Attig, C., & Wessel, D. A personal resource for technology interaction: Development and validation of the Affinity for Technology Interaction (ATI) Scale. *Int. J. Hum. Comput.* **35**, 456–467. <https://doi.org/10.1080/10447318.2018.1456150> (2019).

5. Cerasoli, C. P., Nicklin, J. M. & Nassrelgrgawi, A. S. Performance, incentives, and needs for autonomy, competence, and relatedness: a meta-analysis. *Motiv. Emot.* **40**, 781–813 (2016).

6. He, G., Buijsman, S. & Gadiraju, U. How Stated Accuracy of an AI System and Analogies to Explain Accuracy Affect Human Reliance on the System. *Proceedings of the ACM on Human-Computer Interaction* **7**, 1–29 (2023).

7. Tolmeijer, S., Gadiraju, U., Ghantasala, R., Gupta, A. & Bernstein, A. Second Chance for a First Impression? Trust Development in Intelligent System Interaction. *Proceedings of the ACM Conference on User Modeling, Adaptation and Personalization,* 77–87, doi:10.1145/3450613.3456817 (ACM, 2021).

8. Gaube, S. *et al.* Non-task expert physicians benefit from correct explainable AI advice when reviewing X-rays. *Sci. Rep.* **13**, https://doi.org/10.1038/s41598-023-28633-w (2023).

9. Hoff, T. J. Professional commitment among US physician executives in managed care. *Soc. Sci. Med*. **50**, 1433–1444. <https://doi.org/10.1016/S0277-9536(99)00410-4> (2000).

10. Hekman, D. R., Steensma, H. K., Bigley, G. A., & Hereford, J. F. Effects of organizational and professional identification on the relationship between administrators’ social influence and professional employees’ adoption of new work behavior. *J. App. Psychol.* **94**, 1325–1335. <https://doi.org/10.1037/a0015315> (2009).

11. Gaertig, C., & Simmons, J. P. Do people inherently dislike uncertain advice? *Psychol. Sci*, **29**, 504–520. <https://doi.org/10.1177/0956797617739369> (2018).
